# Adjusted effect size, area under the curve, and c-statistic for evaluating the association between uric acid and mortality in US adults using unweighted and survey-weighted regression, propensity, and prognostic score

**DOI:** 10.7717/peerj.20815

**Published:** 2026-02-19

**Authors:** Shakeel Ahmed, Alok Kumar Dwivedi

**Affiliations:** 1 Division of Biostatistics & Epidemiology, Department of Molecular and Translational Medicine, Texas Tech University Health Sciences Center El Paso, El Paso, TX, United States; 2 Center for Integrated Biostatistics and Epidemiology, Department of Biomedical Informatics, Biostatistics and Medical Epidemiology, University of Missouri School of Medicine, Columbia, Missouri, United States

**Keywords:** Model performance, ROC, PRC, c-statistic, Survey weight, Propensity score, Prognostic score, Cut-point, Stata

## Abstract

Population-based surveys and databases are useful sources for developing prognostic and diagnostic models requiring receiver operating characteristic (ROC) or precision-recall curve (PRC) analyses. The performance of the models is typically summarized with the area under the ROC (rAUC) or PR curves (pAUC) or c-statistic, depending on the study design and analysis. However, these surveys and databases sometimes involve sampling weights due to complex sampling designs. The sampling weights need to be included in the analysis to produce accurate estimates of effect size as well as performance measures. Different types of adjusted analyses, including survey-weighted adjusted analysis, propensity score weight-adjusted (PropSWA), and prognostic score weight-adjusted (ProgSWA) analyses, are typically performed using logistic or Cox regressions as per the study objectives and outcome. We applied these adjusted analyses and compared the effect sizes with or without incorporating sampling weights in the analyses. We explored the relationship between uric acid levels and all-cause mortality in US adults using the National Health and Nutrition Examination Survey dataset, which employs a complex sampling design requiring weight-adjusted analyses. All the models, including unweighted (hazard ratio (HR): 1.09; 95% confidence interval (CI) [1.05–1.12]), survey-weighted (HR: 1.09; 95% CI [1.06–1.12]), unweighted ProgSWA (HR: 1.11; 95% CI [1.07–1.15]), survey-weighted ProgSWA (HR: 1.12; 95% CI [1.09–1.16]), unweighted PropSWA (HR: 1.18; 95% CI [1.12–1.24]), and weighted PropSWA (HR: 1.16; 95% CI [1.10–1.22]) analyses yielded a consistent and positive association between uric acid levels and risk of mortality. These associations were unchanged in various sensitivity analyses. We found marked differences in effect size and predictive performance measures between weighted and unweighted analyses, especially with four categories of uric acid levels. In simulation studies, a survey-weighted propensity model performed better in low-prevalence settings and with skewed exposure. In contrast, survey-weighted prognostic models performed better in high-prevalence settings, particularly with unbalanced exposure and missing data. Our study found a strong association between higher uric acid levels and all-cause mortality in US adults, indicating the importance of proper screening and management of hyperuricemia, particularly in individuals aged >60 years. Based on intensive simulation and real data analyses, we strongly recommend incorporating weights while analyzing studies involving complex sampling designs. Our Stata codes will facilitate analysts to perform a variety of statistical analyses depending on the study objective, presence of confounders, and type of outcomes in survey-weighted data analysis.

## Introduction

Prognostic and diagnostic accuracy of quantitative or categorized markers for a binary outcome or a time-to-event outcome are often needed in clinical and epidemiological research (Ben-Hur et al., 2008; Hendriksen et al., 2013; Horowitz & Savin, 2001). The performance of such prognostic or diagnostic models is generally evaluated using the area under the curve (AUC) with a 95% confidence interval (CI) (Hosmer, Lemeshow & Sturdivant, 2013). Receiver operating characteristic (ROC) curve analysis is the standard tool to estimate the AUC (Ma et al., 2013). The ROC curve portrays an over-optimistic performance of a model when applied to an imbalanced or skewed outcome dataset. Alternatively, the precision-recall curve (PRC) provides better accuracy of the performance of the marker by focusing on under-represented classes in case of imbalanced data among groups (Movahedi, Padman & Antaki, 2023; Saito & Rehmsmeier, 2015). Similarly, Somer’s d and Harrell’s c statistics are used for measuring the predictive performance of the competing models in the analysis of a time-to-event outcome (Graf et al., 1999). These measures are usually reported without proper adjustment of survey weights, which is a crucial step in estimating the effect size (*i.e*., the effect of exposure on an outcome) in association studies or evaluating the accuracy of predictive models in prediction studies when working with complex survey datasets (Kim et al., 2013). The weight-adjusted analysis is also required to estimate the cut point of a quantitative marker in diagnostic studies involving sampling weight.

Furthermore, it is not possible to have a random assignment in the treatment or exposure groups in observational studies, and the groups may differ significantly with respect to the characteristics that could affect the outcome, known as confounding variables. Typically, we need weight-incorporated adjusted estimates of the effect size, AUC, and a threshold for a quantitative marker in the case of diagnostic studies (Janes, Longton & Pepe, 2009; Janes & Pepe, 2008; Pepe, Longton & Janes, 2009). Propensity score weight-adjusted (PropSWA) analysis eliminates these differences by assigning more weight to individuals in the exposed/treatment group who have similar characteristics to those in the unexposed/control group (Ramsey et al., 2019; Stuart, Lee & Leacy, 2013; Yang, 2018). However, the PropSWA approach may not be suitable for optimal bias reduction in estimating the effect size of an exposure due to the positivity assumption, especially for the analysis of a quantitative exposure or multiple exposures (DuGoff, Schuler & Stuart, 2014; Kabata, Stuart & Shintani, 2024; Nguyen & Debray, 2019). In such a situation, a prognostic score weight-adjusted (ProgSWA) approach can be used to adjust confounding variables in the analyses (Hassanzad & Hajian-Tilaki, 2024). Apart from adjusting confounding variables, ProgSWA is also helpful in obtaining a cut point for the quantitative exposure after adjusting for covariates. In most studies, inverse prediction probabilities are used as the weight that adjusts the confounding effect of the covariates and provides an adjusted weighted estimate of effect size after combining the prognostic weight with the survey weight. Multiple types of ProgSWA methods have been discussed and compared using simulation studies (Calkins, 2014). Among different prognostic score balancing methods, the full sample stabilized prognostic score weight adjustment approach has been recommended, which corrects for the selection bias by removing the association between the outcome and the variables inducing the selection bias (Hansen, 2008). In association studies involving complex sampling design, the weight obtained from the propensity or prognostic score analyses and complex survey weights need to be integrated into the statistical analysis for estimating the proper effect size, its 95% CI, and predictive performance measures (Ridgeway et al., 2015; Rubin, 1974). Unfortunately, these practices have been uncommon in routine data analysis of sampling databases, particularly due to the unavailability of user-friendly codes in statistical software. Somer’s d method allows for the estimation of the weight-adjusted AUC with 95% CI and *p*-value. An indirect method, such as obtaining predicted probabilities from a weighted logistic regression model and then calculating sensitivity and specificity or predictive values for each threshold, can be used to construct a ROC or PRC and estimate related AUC (rAUC or pAUC) (Xu et al., 2019). Similarly, these methods can be extended to analyze a time-to-event outcome. We exploited Somer’s d approach to incorporate survey weights and adjust covariates in statistical analyses.

Higher uric acid levels are linked with gout and have been associated with multiple adverse health outcomes, including metabolic and inflammatory diseases (Konta et al., 2020). However, the effect of higher uric acid levels on all-cause mortality in US adults is still unexplored. To address this critical question, we used the National Health and Nutrition Examination Survey (NHANES) database, which involves a complex survey sampling design. Using this dataset, we not only address the unexplored relationship between uric acid levels and all-cause mortality but also provide Stata codes for applying commonly used weighted models in analyzing complex sampling datasets for various types of analyses. The purpose of this study was to (a) determine the pure relationship between uric acid levels and the risk of mortality, with the hypothesis that higher uric acid levels are associated with an increased risk of mortality, and (b) provide computational methods for estimating adjusted effect size, AUC, 95% CI, and associated *p*-value with and without incorporating sampling weight using Stata for analysis of datasets from complex sampling design. This study also aimed to facilitate adjusted analyses after integrating the weights observed from complex survey designs and the weights estimated from PropSWA or ProgSWA. Owing to data generated from an observational study, the association between uric acid levels and the risk of mortality was validated after adjusting for the effects of all covariates on mortality risk through propensity score and prognostic score analyses, with or without incorporating survey/sampling weights. We also extended the methods to obtain Somer’s d and Harrell’s c-statistics for evaluating the accuracy of the quantitative markers for a binary outcome using logistic regression and a survival outcome using Cox regression after adjusting for covariates. In addition, we provided methods for estimating the cut points using survey-weighted adjusted analyses.

## Materials and Methods

As per the evidence-based biostatistics (EBB) practice, it is required that we use preferred approaches of statistical analysis as per the study objective, study design, and data generation process (Dwivedi, 2022; Dwivedi & Shukla, 2020). Accordingly, we performed analyses that accounted for the association objective, requiring the adjustment of confounders and reporting of model performance measures, sampling design requiring accounting for sampling weights in data analysis, and data generation mechanism requiring integration of sampling weights through study design and weights generated through propensity and prognostic score analyses. We discussed different mechanisms for analyzing binary and time-to-event data, incorporating the survey weight and covariate adjustment as per the study design and study objective (Dwivedi, 2022). We developed Stata modules for obtaining rAUC, pAUC for binary variables, and c-statistic for the time-to-event outcome as performance measures under different weights and covariate-adjusted analyses. The suggested modules were then applied to evaluate the association between uric acid levels and all-cause mortality using a population-based NHANES database that requires (i) accounting for survey weight in data analysis (ii) reporting the performance of statistical models for adjusted and predictive models, (iii) estimating the adjusted effect size for the association between uric acid levels and all-cause mortality, and (iv) estimating adjusted prognostic cut point of uric acid for predicting all-cause mortality. Since the outcome was time to all-cause mortality, the primary analyses were conducted using Cox regression models. The performance of the Cox models was summarized with the c-statistic, its 95% CI, and p-value. We further validated the robustness of results by performing adjusted logistic regression analyses. The performance of logistic regression models was measured with rAUC and pAUC, their 95% CIs, and *p*-values. The Cox and logistic regression analyses were conducted in the following six ways (a) unweighted and simple adjusted analysis to adjust for covariates without incorporating survey weight in the analysis, (b) survey-weighted and simple adjusted analysis addressing confounders and survey weight, (c) unweighted PropSWA analysis for covariate adjustment through a propensity score model without incorporating survey weight, (d) survey-weighted with PropSWA analysis for covariate adjustment through propensity score, (e) unweighted ProgSWA analysis for covariate adjustment through a prognostic score model without incorporating survey weight, and (f) survey-weighted with ProgSWA for confounder and selection bias adjustment and estimation of adjusted cut point for uric acid levels using prognostic score approach. In the following sections, we provide the methods and related Stata-based algorithms for computing various statistics, including rAUC, pAUC, and c-statistic for each of the six models.

### Estimation of effect size, rAUC, and pAUC

AUC as a measure of model performance is obtained from a ROC curve (rAUC) using sensitivity and specificity or a PRC (pAUC) using precision and recall values for different thresholds. The Stata users often obtain the AUC using the ***roctab*** command with a given vector of the observed responses on a marker and the outcome (Hanley & McNeil, 1982). It provides an estimated AUC with 95% CI. Similarly, the PRC is obtained from the ***prcurve*** command following ***prtab*** with the same inputs (Cook & Ramadas, 2020). However, the ***prtab*** command only provides the performance measure in terms of pAUC with no confidence limits and *p*-value. Moreover, we often need to include predictors to adjust for confounders or enhance prediction through regression analyses. A logistic regression is often used for analyzing data with a binary outcome and provides the effect size measure in terms of odds ratio (OR). For evaluating the performance of such binary response models, the ***lroc*** command is used as the post-estimation command after **‘*logistic*’ or ‘*logit*’** in Stata, which provides an ROC plot using specificity and sensitivity for different thresholds along with the rAUC value. The ***lroc*** command can accommodate sampling weights to account for complex sampling designs. However, it does not produce comprehensive reporting such as confidence limits and *p*-value of the estimated AUC. In the following subsections, we briefly introduce different analysis scenarios and recommend procedures for obtaining rAUC and pAUC values, 95% CIs, and *p*-values that can accommodate survey weight and adjust covariates. For an outcome variable **y**, an exposure variable **z**, and the covariates **x_1, x_2,…,** the algorithm works as follows:

#### Unweighted and unadjusted analysis, ignoring confounders and sampling weight

The simplest way to perform prognostic or diagnostic analysis is to model the outcome with the exposure by ignoring confounding variables and survey weights. Such a situation may apply to randomized trials where the adjustments to covariates are made through randomization at the design stage of the experiment. For a binary outcome, a logistic regression is recommended, and the analysis can be performed using the following algorithm:

#### Unweighted and adjusted analysis

Observational studies conducted using simple random designs require only the adjustment of confounders without incorporating survey weights. The computation of adjusted effect size, rAUC, and pAUC can be performed using Algorithm 1 with modification in Step **I,** *i.e*., by replacing ***logistic y z with logistic y z x_1 x_2…*,** where ***x_1 x_2…*** are the covariates to be adjusted.

**Algorithm 1:** Stata algorithm for computing effect size, rAUC, pAUC, 95% CI, and *p*-value using unweighted and unadjusted logistic regression.

**I.** Fit a model with ***logistic y z*** and obtain the predicted probabilities *as* ***predict p_hat*,** where ***z*** is the exposure variable that can be categorical or continuous.
**II.** Use **the *senspec*** command as ***senspec y p_hat, sensitivity(sens) specificity(spec) fpos(fpos) ntpos(pos) nfpos(nfpos)***, *w*here ***sens, spec, fpos, pos***, and ***nfpos*** refer to the sensitivity, specificity, proportion of false positives, number of true positives, and the number of false positives, respectively.
**III.** The rAUC is obtained as ***gen auc=r(integral)*** followed by ***integ sens fpos, trapezoid*.**
**IV.** Using frequency distribution of **y** with ***tabulate y, matcell(d)*,** find number of positive cases ***n_pos*** and negative cases ***n_neg*** and obtain standard error of AUC as se_auc=sqrt((auc*(1-auc)+(n_pos-1)(q_1-auc^2)+(n_neg-1)(q_2-auc^2))/(n_pos*n_neg)), where ***q_1=auc/(2-auc)*** and q_2=2*auc^2/(1+auc) are two scalars. The mathematical details of the standard error formula can be found in a published article (Hanley & McNeil, 1982).
**V.** The lower and upper confidence limits are generated as gen ll_auc= auc-1.96*se_auc, and gen ul_auc= auc+1.96*se_auc, respectively. Assuming asymptotic normality, we can compute the ***p_value*** as **gen z=(auc-0.5)/se_auc **with gen p_value=2*(1-normal(abs(z))). Finally, the results are tabulated using the command ***tabstat auc ll_auc ul_auc p_value*.**
**VI.** Plot an ROC curve using**: *twoway (scatter sens fpos, sort(sens fpos) connect(L) mlab()) (line sens* sens)**. For further details, please see Tibbles & Melse (2023).
**VII.** The results for pAUC are obtained with the same steps, only by replacing the **auc** formula in Step **III** as ***integ ppv sens, a trapezoid*** with ***gen prc=r(integral)*.**

#### Survey-weighted and unadjusted analysis

In the case of a complex sampling design, we can incorporate the survey weights in the analysis for the situation described in (**a**) *i.e*., when there is no influence of confounding variables in the analysis. For unadjusted survey-weighted analysis, the rAUC, pAUC, and related details are obtained using the following algorithm:

#### Survey-weighted and adjusted analysis

In most of the association analyses with data obtained from complex survey designs, it is recommended to incorporate both the survey weights as well as critical covariates in the model to find the adjusted association of the exposure with the outcome (Dwivedi, 2022). The computation of adjusted effect size, rAUC, pAUC, and related measures can be performed using Algorithm 2 with modification in Step **II,** *i.e*., replacing **svy: *logistic y z with* svy: *logistic y z x_1 x_2…*** where ***x_1, x_2…*** are the covariates to be adjusted.

**Algorithm 2:** Stata algorithm for computing effect size, rAUC, pAUC, 95% CI, and *p*-value using survey-weighted and unadjusted logistic regression.

**I.** The survey set command is used with specific details on stratification and stages such as ***svyset psu [pw=survey_weight], strata(strata_var) singleunit(certainty)***, where ***psu*** identifies the primary sampling units, ***survey_weight*** is the survey weight, and ***strata_var*** is the stratification variable.
**II.** Fit a logistic model as **svy**: ***logistic y z*** and obtain the predicted probabilities *as* ***p_hat*,** where ***z*** is the exposure variable that can be categorical or continuous.
**III.** Follow Steps **II–VII** of Algorithm 1 for the rest of the analysis.

#### Survey-weighted and PropSWA analysis for binary exposure

One way to adjust for covariates is to incorporate the confounding variables in the weights by computing the propensity score weight and integrating this weight with the survey weight if the exposure is a binary variable. Such covariate adjustment may be helpful when there is a small sample size study, causing high dimensionality issues, or when it requires the removal of confounding variables in observational interventional studies (Dwivedi, 2022). For a logistic model, propensity weights are obtained using the following steps in Stata:

#### Survey-weighted PropSWA analysis for continuous exposure

In case of continuous exposure, Steps **II and III of **Algorithm 3 **are** updated as follows:

**I.** Obtain residuals, predicted values, and standard deviation of residuals after running a multiple linear regression: ***reg Z u_1, u_2,…u_k*** *with*
***predict residuals, resid***
***predict muhat, xb***
***sum residual***
***local sdhat=r(sd)***
***The sum Z and obtain the score***
gen gps = (1/(‘sdhat’*sqrt(2*_pi))) * exp(-((residual)^2)/(2*‘sdhat’^2))
**II.** Obtain the same score following Step I without including the covariates in the model (null model) as follows
***sum y***
***local muhat0 =r(mean)***
***local sdhat0 =r(sd)***
gen gps0 = (1/(‘sdhat0’*sqrt(2*_pi))) * exp(-((y - ‘muhat0’)^2)/(2*‘sdhat0’^2))

**Algorithm 3:** Stata algorithm for computing effect size, rAUC, pAUC, 95% CI, and *p*-value using survey-weighted and PropSWA logistic regression.

**I.** Obtain propensity score (***P_score***) using the command ***(pscore Z u_1 u_2…u_k, P_score) logit***, where ***z*** is a binary exposure or treatment indicator, and ***u_1, u_2,…u_k*** are k confounding variables to be included for adjustment.
**II.** The propensity score weight is then obtained using the inverse probability treatment weight (iptwt) method as **gen iptwt=(z/*P_score*)+((1-z)/(1- *P_score*)) **with the sum of weights ***egen sumofweights = total(iptwt), and*** normalized weights ***gen norm_weights = iptwt/sumofweights*.**
**III.** The final weight after combining with the survey weight, rounding to the nearest whole number, is obtained as gen prop_weight= round(norm_weights*survey_weight).
**IV.** Follow Steps **I–III** of Algorithm 2 after replacing ***survey_weight*** with ***prop_weight*** for computing effect size, rAUC, pAUC, 95% CI, and *p*-value.

*Generate the ratio as* ***gen iptwt = gps0/gps*** with the sum of weights ***egen sumofweights = total(iptwt), and*** normalized weights ***gen norm_weights = iptwt/sumofweights*.** Follow Steps **I–III** of Algorithm 2 after replacing ***survey_weight*** with ***norm_weights*** for computing effect size, rAUC, pAUC, 95% CI, and *p*-value.

#### Survey-weighted ProgSWA analysis

An alternative to propensity score-adjusted analysis, prognostic score-adjusted analysis can also be performed for dealing with high-dimensional data in the analysis, removing the confounding effects in observational interventional studies, or determining the adjusted cut point of a marker in diagnostic studies (Hajian-Tilaki, 2018). The prognostic score is obtained as a full sample stabilized prognostic score approach, as discussed in an article (Hansen, 2008). Thereafter, the weights derived from prognostic score analysis can be combined with survey weights to obtain a final weight that incorporates survey weights and adjusts for confounding variables as well as selection bias. The survey weighted with ProgSWA analysis can be performed using the following algorithm:

**Algorithm 4:** Stata algorithm for computing effect size, rAUC, pAUC, 95% CI, and *p*-value using survey-weighted and ProgSWA logistic regression.

**I.** Obtain predicted outcomes using gen var =exp(l_p)/(1+exp(l_p)), where l_p= _b[cons]+_b[Z*0]+_*after considering exposure status at zero;* ***predict var*** as a post-estimation command to logistic ***y z u_1 u_2…***, where ***u_1, u_2,…u_k*** are the covariates to be adjusted in the analysis and z is the exposure.
**II.** Compute prognostic score *as Pr_score*=mean(y)*y/*var* +(1- mean(y))*(1-y)/(1- *var*).
**III.*** Obtain* final weight after combining the score obtained in Step **II** with the survey weight, rounding to the nearest whole number as gen prog_weight = round(Pr_score *survey_weight)
**IV.** Follow Steps **I–III** of Algorithm 2 after replacing ***survey_weight*** with **prog_weight **for computing effect size, rAUC, pAUC, 95% CI, and *p*-value.

### Estimation of a cut point of a continuous exposure

One of the major objectives of diagnostic modeling is to obtain a cut point of continuous markers, incorporating survey sampling design in data analysis. When data is obtained from complex surveys, then there is a likelihood of obtaining multiple confounding variables which need to be incorporated in the analyses. Moreover, the incorporation of weight and covariate adjustment is required while obtaining the cut point. A variety of methods are available for obtaining cut points of continuous exposure based on different performance measures, such as minimum distance and Youden’s index, *etc*. (Bantis, Nakas & Reiser, 2014; Hajian-Tilaki, 2018). However, no proper Stata module is available to obtain a cut point that makes proper incorporation of survey weight after adjusting for the confounding variables. Based on sensitivity (***sens***) and specificity (***spec***) using ***senspec*** (see Step **II** Algorithm 1 for unadjusted analysis or Algorithm 4 for ProgSWA), we obtain the cut point for ***z*** (a continuous exposure) with the following Stata codes:

***gen youdenid= sens-(1-spec)***

***egen youdenidmax= max(youdenid)***

***list sens spec youdenidmax z if abs(youdenid -youdenidmax)<0.000001***

### Estimation of Harrell’s c statistic

Harrell’s c-statistic (concordance index) (Harrell et al., 1982) is an alternative performance measure for estimating the proportion of concordance pairs between observed and predicted outcomes. Somer’s d method for obtaining the c-statistic in Stata for the case of binary response modeling with and without incorporating survey or propensity weights has been described (Newson, 2002). The confidence limits of the c-statistic can be obtained as described in a published article (Newson, 2006). A recent update (Newson, 2020) on the ***somersd*** is illustrated as follows. For a binary outcome, the ***somersd*** command is used as ***somersd y y_hat*, *tr(transformation_name)*** to obtain Harrell’s c-statistic as a measure of the predictive performance, where ***y_hat*** refers to the predicted values of the binary variable ***y*,** and the ***tr( )*** command specifies that the estimates are to be transformed using a specific method, readers can find detail on specific transformations from a published article (Newson, 2002). Another way is to use the predicted probabilities ***P_hat*** instead of ***y_hat*** for the same purpose. The survey or prognostic weight can be adjusted using the **pw** command as ***somersd y y_hat* [pw=weight_name], *tr(transformation_name)*,** where y_hat is the predicted responses from a weight-adjusted model. The ***somersd*** command produces the detailed output, including the c-statistic, 95% confidence limits, and the *p*-value.

In addition, Harrell’s c-statistic is widely used as the measure of concordance for the time-to-event or survival outcome analysis. The c-statistic can be obtained after the **stcox** command using the post-estimation command ***estat concordance***. For a time-to-event variable ***t*** with ***d*** event indicator, ***z*** exposure (or treatment indicator), and ***x_1, x_2…x_k***, the ***k*** covariates, the Cox regression can be developed with **stcox z *x_1, x_2…x_k*** followed by ***stset t, failure (d=1)***. Although the ***estat concordance*** command produces detailed results on the c-statistic, it does not incorporate any type of weight, which may lead to produce inaccurate estimates. By mentioning other limitations of ***estat concordance***, a study (Newson, 2010) suggested using the ***somersd*** command as a post-estimation command by using the inverse predicted hazards. We provide algorithms for obtaining the c-statistic for six different scenarios (discussed in ‘Estimation of effect size, rAUC, and pAUC’) in this section:

#### Unweighted and unadjusted Cox regression analysis

The straightforward way to perform a Cox regression analysis is to simply model the outcome (time-to-event) with the exposure by ignoring confounding variables as well as the survey weights.

The ***tdist*** option is used to obtain confidence limits and *p*-value using the Student’s t distribution. Note that the **z** used in ***transf(*z*)*** *is* different from the ***z*** used as the exposure variable. We used the time-to-event variable ***t*** and the survival sample indicator ***st*** *(*obtained in ***stset****)* to estimate rank parameters of the inverse hazard ratio with respect to ***t*** (censored by ***censind***). The ***somersd*** command produces detailed results, including confidence limits and p-values for both statistics.

#### Unweighted and adjusted Cox regression analysis

Studies with a simple random design require only adjustment of confounders without survey weight. The computation of the c-statistic in such analysis can be performed using Algorithm 5 with modification in Step **I,** *i.e*., replacing ***stcox z with stcox z x_1 x_2…*,** where ***x_1 x_2…*** are the covariates to be adjusted.

**Algorithm 5:** Unweighted and unadjusted estimation of Harrell’s c-statistic using predicted inverse hazard.

**I.** Run the **stcox z** command after the ***stset*** command with the exposure variable ***z*.**
**II.** Obtain predicted hazard as ***predict hr*** and find inverse hazard as ***gen invhr=1/hr*.**
**III.** Use the ***inhvr*** to obtain the c-statistic as ***somersd* _t invhr if _st==1, cenind(censind) tdist transf(c),** *where* ***censind*** is the censoring indicator obtained as ***gen censind=1-_d if _st==1 and _d*** is the event indicator. The ***transf (c)*** and ***transf(*z*)*** are used to obtain the ***c*** and ***d*** statistics, respectively.

#### Survey-weighted and unadjusted Cox regression analysis

In the case of complex surveys, we can incorporate the survey weights in the analysis for the situation described in 2.1 **(a)** *i.e*., when there is no influence of confounding variables in the analysis. For survey-weighted Cox regression, the computation of the c-statistic is done by incorporating the ***survey_weight*** in ***somersd*** command used in Step **III** of Algorithm 5 as ***somersd _t invhr [pw=survey_weight] if _st==1, cenind(censind) tdist transf(c)*** followed by ***svy:stcox z*** command.

#### Survey-weighted and adjusted Cox regression analysis

The computation of the c-statistic is performed using Algorithm 6 for survey-weighted analysis with adjustment of covariates:

**Algorithm 6:** Adjusted survey-weighted estimation of Harrell’s c-statistic using predicted inverse hazard.

**I.** Run a Cox regression using **svy**: **stcox z x_1 x_2….** command after the ***stset*** command with the exposure variable ***z*** and set of covariates **x_1 x_2….**
**II.** Obtain predicted hazard as ***predict hr*** and find inverse hazard as ***gen invhr=1/hr*.**
**III.** Use the ***inhvr*** with ***somersd*** to obtain the c- statistic as ***somersd* _t invhr if _st==1, cenind(censind) tdist transf(c),** *where* ***censind*** is the censoring indicator obtained as ***gen censind=1-_d if _st==1*.** The ***transf (c)*** and ***transf(*z*)*** are used to obtain the ***c*** and ***d*** statistics.

#### Survey-weighted with PropSWA Cox regression analysis

For Cox regression analysis, a final weight denoted by ***prop weight*** is obtained by combining propensity weight and survey weight using Steps **I–III** of Algorithm 3. The final weight can be adjusted with the ***somersd*** command used in Step **III** of Algorithm 5 as ***somersd _t invhr [pw=prop_weight] if _st==1, cenind(censind) tdist transf(c)***.

#### Survey-weighted with ProgSWA Cox regression analysis

For Cox regression analysis with ProgSWA, the final weight, *i.e*., ***prog_weight*** is obtained using Steps **I-II** of Algorithm 4. The final weight can then be adjusted with the ***somersd*** command used in Step **III** of Algorithm 5:

***somersd _t invhr [pw=prog_weight] if _st==1, cenind(censind) tdist transf(c)***.

### Data analysis

The data from NHANES, conducted by the U.S. Centers for Disease Control and Prevention, a nationally representative health survey of noninstitutionalized U.S. civilians, was used to evaluate the association between uric acid levels and all-cause mortality. We included seven cycles from 2005 to 2018, *i.e*., NHANES 2005-06, 2007–08, 2009–10, 2011–12, 2013–14, 2015–16, and 2017–18. The follow-up time for mortality status was derived from the date of the survey to the year of death or the end of the mortality follow-up date, *i.e*., December 31, 2019 (Johnson et al., 2013). The data on uric acid concentrations (in mg/dL) as a primary exposure were included in all the statistical analyses. The concentration of uric acid in serum above the threshold level of 7 mg/dL in men and >6 mg/dL in women was considered hyperuricemia (Borghi & Piani, 2021) and included in sensitivity analyses. The baseline covariates included respondent’s age (in years), marital status (never married, married, living with a partner, other categories, and unknown), sex (male *vs*. female), education (lower than high school, high school diploma or general equivalency diploma, some college or AA degree, above college qualification, unknown), income (less than $45,000, $45,000 to $99,999, $ 100,000 or above, Unknown), ethnicity (Hispanic, non-Hispanic, non-Hispanic black, other racial groups), smoking status (smoker, non-smoker, unknown), alcohol usage (yes, no, unknown), body mass index (BMI, kg/m^2^)(<25, 25 to 30, 30 to 35, >35) and physical activity (yes, no) according to Global Physical Activity Questionnaire. We obtained data on *N* = 37,742 complete cases after excluding the non-adult individuals (age <18), and cases with missing data on uric acid levels or mortality status.

To determine the effect size for the association between uric acid concentrations and mortality, we performed logistic regression and Cox regression analyses. Prior to applying Cox proportional models, the proportionality assumption was tested using the Schoenfeld test and its residual plot. In addition, we tested the assumption of inverse probability treatment, and accordingly, a strong prognostic variable, such as age, was not included in generating the prognostic score, whereas it was adjusted for in the regression analysis. Similarly, gender was not included in generating the propensity score, while it was adjusted in the regression analysis. We computed rAUC, pAUC, and c-statistic for all these models. The findings from primary analyses were further confirmed by including binary and categorized forms of uric acid levels using Cox and logistic regression models through multiple sensitivity analyses. We further validated the performance of these models in older individuals aged >60 years. We obtained adjusted cut points of uric acid concentrations in relation to mortality using unweighted analysis, survey-weighted analysis without adjusting for covariates, and ProgSWA using Youden’s method as discussed in ‘Estimation of a Cut Point of a Continuous Exposure’. All statistical analyses were performed using Stata 17.0. The Stata codes for real data analysis are provided in the Supplemental Material (Appendix A). A flowchart summarizes various primary and sensitivity analyses (Fig. S1).

**Figure 1:**
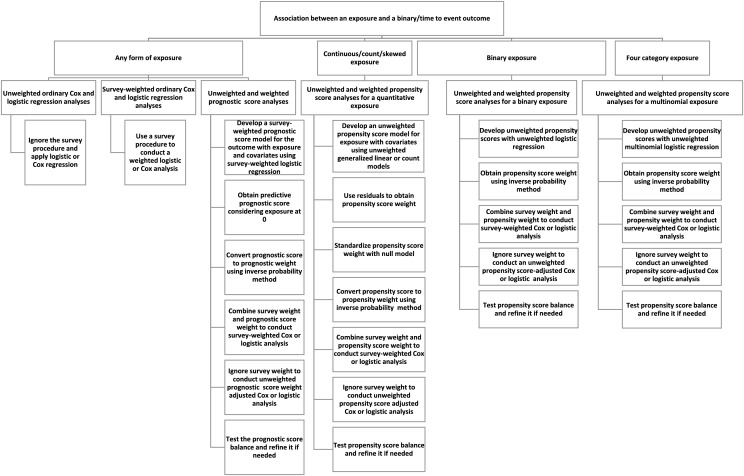
Step-by-step instructions for conducting various types of analysis based on the type of exposure variable.

We compared the performance of various methods, including unweighted and weighted survey-adjusted analyses, propensity score, and prognostic score analyses using simulation studies. A flowchart summarizes different scenarios and comparisons across modules (Fig. 1)

We followed the steps outlined by DuGoff, Schuler & Stuart (2014) and Ridgeway et al. (2015) for generating data. Accordingly, a binary outcome variable (y), a binary exposure variable (t), and a normally distributed covariate (x) were generated on a stratified population size of 90,000. The population consisted of three strata of equal size, each with different selection probabilities. We generated data in such a way that the confounder (x~strata) was associated with the stratum, exposure allocation was associated with the covariate (t~x), and the outcome (y~t, x) was associated with the confounder as well as exposure, and the selection probability was also associated with an exposure and a covariate (s~t, x). We simulated population data for two scenarios: one with a low outcome prevalence (~15%) and another with a common outcome prevalence (~40%). For each scenario, we randomly selected sample sizes of 500, 1,000, and 2,000 using stratified sampling with an unequal probability selection method. The percent bias in the adjusted effect size of exposure, the 95% confidence interval width of the exposure coefficient, the coverage probability of the true coefficient within the estimated 95% CI, the percent bias in the estimated AUC, and the coverage probability of the true AUC were compared across methods. In common prevalence studies, we also compared the performance of different analysis strategies in the presence of a count data exposure generated using a Poisson distribution, a skewed exposure generated using a gamma distribution, an unbalanced exposure (with a proportion of 15%), and missing data (20%) on a covariate/confounder.

## Results

Among 37,742 individuals selected for final analysis with a mean age of 47.9 years, 6,841 (17.4%) had elevated uric acid with an overall average of 5.43 mg/dL. There were 3,826 (7.6%) mortalities recorded during a total of 180 months of follow-up period. The distribution of baseline characteristics is displayed in Table S1.

### Unadjusted and adjusted associations between uric acid levels and mortality with or without accounting for survey weights

The survey-weighted (HR: 1.09; 95% CI [1.06–1.12]) and unweighted (HR: 1.09; 95% CI [1.05–1.12]) analyses yielded the same effect size for the association between uric acid levels and risk of mortality. Although there were no differences in the risk of mortality associated with uric acid levels between survey-weighted ProgSWA (HR: 1.12; 95% CI [1.09–1.16]) and unweighted ProgSWA (HR: 1.11; 95% CI [1.07–1.15]) analyses, a slightly higher effect size was obtained with survey-weighted PropSWA (HR: 1.16; 95% CI [1.10–1.22]) and unweighted PropSWA (HR: 1.18; 95% CI [1.12–1.24]) (Table 1). These findings were consistently observed in survey-weighted or unweighted logistic regression analyses, after adjusting for all covariates (Table 1) or adjusting for covariates without considering BMI (Table S2). Furthermore, the pattern of association remained consistent and significant even when analyzed in a binary form for uric acid levels across all models (Table S3).

**Table 1:** Association between uric acid (continuous) and all-cause mortality (NHANES 2005–2018).

	Cox regression		Logistic regression	
Association models	HR (95% CI)	*p*-value	OR (95% CI)	*p*-value
Unweighted & adjusted analysis	1.09 [1.06–1.12]	<0.0001	1.13 [1.10–1.17]	<0.0001
Survey-weighted adjusted analysis	1.09 [1.05–1.12]	<0.0001	1.12 [1.08–1.16]	<0.0001
Unweighted PropSWA analysis	1.18 [1.12–1.24]	<0.0001	1.20 [1.13–1.27]	<0.0001
Survey-weighted & PropSWA analysis	1.16 [1.10–1.22]	<0.0001	1.18 [1.12–1.25]	<0.0001
Unweighted ProgSWA analysis	1.12 [1.09–1.16]	<0.0001	1.17 [1.12–1.21]	<0.0001
Survey-weighted & ProgSWA analysis	1.11 [1.07–1.15]	<0.0001	1.17 [1.10–1.23]	<0.0001

**Note:**

Adjusted models included age, gender, income, education, ethnicity, marital status, body mass index, smoking, alcohol use, and physical activity; PropSWA, Propensity score weight-adjusted; gender was not included in creating propensity score weight but was adjusted in regression analysis; age was not included in creating prognostic score weight but was adjusted in regression analysis. ProgSWA, Prognostic score weight-adjusted; OR, Odds ratio; HR, Hazard ratio; CI, Confidence interval.

Although no differences were noted in the unweighted and weighted models with continuous (Table 1) and binary (Table S3) forms of uric acid levels in the analyses, marked differences were observed after considering categorized uric acid levels in the analyses (Table S4). Both second quartile (HR: 1.15; 95% CI [1.02–1.30], *p* = 0.024) and fourth quartile (HR: 1.42; 95% CI [1.26–1.61], *p* < 0.001) of uric acid levels were associated with higher risk of mortality in survey-weighted analyses whereas only fourth quartile (HR: 1.39; 95% CI [1.25–1.54], *p* < 0.001) of uric acid levels was associated with higher risk of mortality in unweighted analysis. Moreover, higher levels of uric acid levels including second (HR: 1.50; 95% CI [1.14–1.99] *vs* HR: 1.17; 95% CI [1.05–1.30]), third (HR: 1.54; 95% CI [1.08–2.20] *vs* HR: 1.15; 95% CI [1.04–1.28]) and fourth quartile (HR: 1.73; 95% CI [1.37–2.19] *vs* HR: 1.62; 95% CI [1.45–1.82]) were associated with an increased risk of mortality in survey-weighted prognostic models to a greater extent than in survey-weighted propensity models (Table S4).

The risk of mortality associated with hyperuricemia was consistently observed in older individuals, with no differences in the effect size between the unweighted analysis (HR: 1.31; 95% CI [1.21–1.42]) and the survey-weighted analysis (HR: 1.33; 95% CI [1.19–1.48]). Contrary to the continuous form of uric acid levels in the analyses, prognostic score models (unweighted HR: 1.52; 95% CI [1.35–1.71] and weighted HR: 1.48; 95% CI [1.26–1.73]) produced higher effect sizes compared to propensity score models (unweighted HR: 1.27; 95% CI [1.17–1.38] and weighted HR: 1.25; 95% CI [1.12–1.39]). These findings were consistently observed with survey-weighted or unweighted logistic regression analyses (Table 2). Residual plots (Fig. S2) and no changes in results between logistic and Cox regression confirmed the absence of a violation of the proportionality assumption in Cox models.

**Table 2:** Association between uric acid level and all-cause mortality among individuals aged >60 years (NHANES 2005–2018).

	Cox regression		Logistic regression	
Association models	HR (95% CI)	*p*-value	OR (95% CI)	*p*-value
Unweighted & adjusted analysis	1.31 [1.21–1.42]	<0.0001	1.46 [1.30–1.63]	<0.0001
Survey-weighted adjusted analysis	1.33 [1.19–1.48]	<0.0001	1.41 [1.22–1.63]	<0.0001
Unweighted PropSWA analysis	1.27 [1.17–1.38]	<0.0001	1.56 [1.42–1.71]	<0.0001
Survey-weighted PropSWA analysis	1.25 [1.12–1.39]	0.0001	1.27 [1.10–1.45]	0.0008
Unweighted ProgSWA analysis	1.52 [1.35–1.71]	<0.0001	1.55 [1.37–1.76]	<0.0001
Survey-weighted & ProgSWA analysis	1.48 [1.26–1.73]	<0.0001	1.46 [1.24–1.73]	<0.0001

**Note:**

Adjusted models included age, gender, income, education, ethnicity, marital status, body mass index, smoking, alcohol use, and physical activity; gender was not included in creating propensity score weight but was adjusted in regression analysis; age was not included in creating prognostic score weight but was adjusted in regression analysis. PropSWA, Propensity score weight-adjusted; ProgSWA, Prognostic score weight-adjusted; OR, Odds ratio; HR, Hazard ratio; CI, Confidence interval.

### Model performance measures for the association between uric acid levels and mortality, with or without accounting for survey weights

There were no differences in model accuracy as measured by c-statistic or rAUC. The predictive accuracy of the model with uric acid concentrations only was found to be similar between unweighted (rAUC = 89%) and weighted (rAUC = 88%) analyses. Similarly, after eliminating the effects of confounding variables, the rAUC was observed to be consistent across models, including PropSWA (weighted rAUC = 0.54 *vs* unweighted rAUC = 0.55) and ProgSWA(weighted rAUC = 0.54 *vs* unweighted rAUC = 0.55) (Table 3). Contrary to the c-statistic or rAUC, the estimated pAUC was found to be different in survey-weighted (pAUC = 0.49) and unweighted (pAUC = 0.55) analyses. Furthermore, unweighted (pAUC = 0.25) and weighted (pAUC = 0.24) prognostic score models yielded similar and higher pAUC than unweighted (pAUC = 0.17) and weighted (pAUC = 0.12) propensity score models.

**Table 3:** Summary of performance measures (c-statistic and rAUC) of different models.

	Performance measure	Estimate	95% CI	
Unweighted & adjusted analysis	c-statistic	0.75	[0.74–0.76]
	rAUC	0.89	[0.88–0.89]
	pAUC	0.55	[0.54–0.56]
Survey-weighted adjusted analysis	c-statistic	0.77	[0.75–0.78]
	rAUC	0.88	[0.87–0.89]
	pAUC	0.49	[0.48–0.49]
Unweighted PropSWA analysis	c-statistic	0.53	[0.51–0.56]
	rAUC	0.55	[0.54–0.56]
	pAUC	0.17	[0.16–0.17]
Survey-weighted & PropSWA analysis	c-statistic	0.58	[0.55–0.60]
	rAUC	0.54	[0.53–0.55]
	pAUC	0.12	[0.12–0.13]
Unweighted ProgSWA analysis	c-statistic	0.55	[0.52–0.58]
	rAUC	0.55	[0.54–0.56]
	pAUC	0.25	[0.24–0.26]
Survey-weighted & ProgSWA analysis	c-statistic	0.53	[0.48–0.59]
	rAUC	0.54	[0.53–0.55]
	pAUC	0.24	[0.23–0.25]

**Note:**

Adjusted models included age, gender, income, education, ethnicity, marital status, body mass index, smoking, alcohol use, and physical activity; gender was not included in creating propensity score weight but was adjusted in regression analysis; age was not included in creating prognostic score weight but was adjusted in regression analysis. PropSWA, propensity score weight-adjusted; ProgSWA, prognostic score weight-adjusted; rAUC, area under ROC curve; pAUC, area under ROC curve; CI, confidence interval; ROC, receiver operating characteristic curve.

### Unadjusted and adjusted cut points with or without survey weights

The adjusted cut point for uric acid concentrations was estimated to be 4.6 mg/dL using ProgSWA analysis as compared to 5.6 mg/dL for unweighted analysis, and 6 mg/dL for survey-weighted analysis without adjusting for covariates. The cut-point was observed relatively higher for elderly subjects with 7.3 mg/dL using ProgSWA analysis, 6.8 mg/dL using unweighted analysis, and 6 mg/dL using survey-weighted analysis without adjusting for covariates (Table 4).

**Table 4:** Cut point of uric acid in relation to mortality using unadjusted and adjusted models.

Analysis	Cut-point (entire cohort)	Cut-point (age >60)
Unweighted & unadjusted analysis	5.6	6.8
Survey-weighted & unadjusted analysis	6	6
Survey-weighted & ProgSWA analysis*	4.6	7.3

**Notes:**

PropSWA, Propensity score weight adjusted; ProgSWA, prognostic score weight-adjusted.

* Adjusted model included age, gender, income, education, ethnicity, marital status, body mass index, smoking, alcohol use, and physical activity.

### Simulation studies

In simulation studies with a binary exposure and a continuous confounder, negligible differences were observed across models for different scenarios, except for propensity score models, which tended to yield slightly more biased estimates. However, survey-weighted analyses yielded consistently better performance in terms of bias, coverage probability in the estimated effect size, and the estimated AUC compared to unweighted analyses. Survey-weighted propensity score model outperformed others for low-prevalence studies across different sample sizes, considering both coefficient and model performance estimates. In contrast, the survey-weighted prognostic score model produced better performance relative to other models for high-prevalence studies across different sample sizes (Table 5).

**Table 5:** Simulated results for estimated treatment effect size and area under the curve for different prevalence and sample sizes.

	Low prevalence					High prevalence					
	Effect size			AUC		Effect size				AUC	
	Bias	Coverage probability	Width of 95% CI	Bias	95% CI Coverage	Bias		Coverage probability	Width of 95% CI	Bias	Coverage probability
*N* = 500											
UW	0.341	0.954	0.314	0.643	0.941	**−0.197**		**0.946**	**0.207**	**−0.004**	**0.959**
SW	**0.380**	**0.949**	**0.328**	**0.490**	**0.948**	0.160		0.949	0.217	0.391	0.950
UWPropSWA	2.338	0.957	0.318	−0.135	0.956	3.958		0.952	0.214	0.792	0.956
SW&PropSWA	**0.499**	**0.953**	**0.329**	**−0.134**	**0.946**	2.186		0.958	0.218	0.939	0.953
UWProgWA	1.135	0.954	0.335	−0.712	0.943	−0.138		0.948	0.208	0.600	0.948
SW&ProgSWA	1.375	0.954	0.317	0.073	0.952	**0.117**		**0.951**	**0.219**	**−0.106**	**0.950**
*N* = 1,000											
UW	−0.319	0.960	0.218	0.332	0.954	−0.239		0.950	0.146	−0.221	0.950
SW	−0.186	0.957	0.228	0.206	0.954	**0.101**		**0.949**	**0.152**	**0.133**	**0.944**
UWPropSWA	1.628	0.960	0.221	−0.180	0.961	3.812		0.940	0.150	0.837	0.949
SW&PropSWA	**−0.101**	**0.953**	**0.229**	**−0.126**	**0.957**	2.028		0.945	0.153	0.964	0.937
UWProgWA	0.211	0.964	0.231	−0.745	0.944	−0.276		0.949	0.146	0.647	0.950
SW&ProgSWA	**0.149**	**0.963**	**0.219**	**−0.100**	**0.959**	**0.010**		**0.954**	**0.153**	**−0.045**	**0.956**
*N* = 2,000											
UW	−0.290	0.956	0.153	0.262	0.949	−0.270		0.951	0.103	−0.345	0.941
SW	0.013	0.957	0.160	0.163	0.947	**0.132**		**0.960**	**0.107**	**0.066**	**0.963**
UWPropSWA	1.610	0.958	0.155	−0.119	0.957	3.703		0.932	0.106	0.846	0.944
SW&PropSWA	**0.101**	**0.959**	**0.161**	**0.024**	**0.960**	2.045		0.946	0.108	1.013	0.920
UWProgWA	−0.059	0.960	0.162	−0.723	0.945	−0.323		0.951	0.103	0.686	0.941
SW&ProgSWA	**−0.024**	**0.952**	**0.153**	**−0.091**	**0.953**	**0.049**		**0.958**	**0.108**	**0.006**	**0.960**

**Note:**

UW, Unweighted; SW, Survey-weighted; PropSWA, Propensity score weight-adjusted; ProgWA, Prognostic score weight-adjusted; AUC, Area under curve; CI, Confidence interval; bold highlighted are the values for the two best performed models

**Figure 2:**
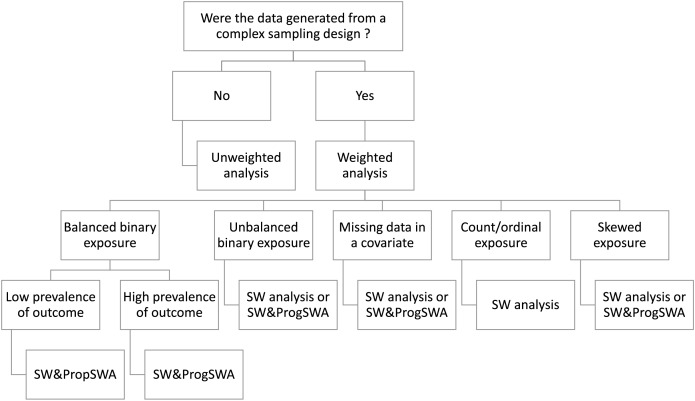
Simulation and real data analysis-based recommendations for conducting various types of analysis based on the type of exposure variable and missing data on covariates. UW, Unweighted; SW, survey-weighted; PropSWA, propensity score weight-adjusted.

Compared to models with a binary exposure, unweighted analyses yielded biased estimates of the coefficient and AUC, particularly when the exposure was continuous and skewed, data on a confounder were missing, or the exposure was unbalanced. In all scenarios, including count exposure, skewed exposure, unbalanced exposure, and missing data in a confounder, ordinary survey weighted analysis produced better performance than alternatives. Survey-weighted prognostic score model also produced better performance for unbalanced exposure or with missing data compared to propensity score models. However, propensity score models with skewed exposure yielded good performance relative to prognostic score models (Table 6). Based on intensive simulation studies and real data analysis, we recommended the selection of appropriate weighted data analysis (Fig. 2).

**Table 6:** Simulated results for estimated treatment effect size and area under the curve for different types of exposures and missing data on a confounder.

	Effect size			AUC	
	Bias	Coverage probability	Width of 95% CI	Bias	Coverage probability
**Count exposure**					
UW	0.077	0.963	0.054	1.782	0.529
SW	**0.025**	**0.945**	**0.073**	**−0.008**	**0.939**
UWPropSWA	0.651	0.946	0.114	−32.07	0.083
SW&PropSWA	0.257	0.965	0.068	−32.44	0.000
UWProgWA	0.269	0.946	0.055	−9.47	0.071
SW&ProgSWA	0.221	0.943	0.055	−9.501	0.079
**Continuous skewed exposure**						
UW	−1.388	0.955	0.063	3.057	0.113
SW	**−0.161**	**0.959**	**0.078**	**0.031**	**0.963**
UWPropSWA	1.723	0.943	0.078	−8.389	0.019
SW&PropSWA	**1.196**	**0.943**	**0.078**	**0.768**	**0.946**
UWProgWA	−1.088	0.959	0.063	−10.903	0
SW&ProgSWA	−1.131	0.958	0.063	−10.920	0
**Unbalanced exposure**					
UW	1.368	0.958	0.127	1.756	0.675
SW	**0.311**	**0.964**	**0.132**	**0.027**	**0.958**
UWPropSWA	3.869	0.932	0.130	−8.136	0.002
SW&PropSWA	1.759	0.961	0.145	−8.451	0.018
UWProgWA	2.051	0.955	0.130	−5.441	0.037
SW&ProgSWA	**0.951**	**0.961**	**0.128**	**0.097**	**0.962**
**Missing data in a confounder**						
UW	0.680	0.961	0.123	1.405	0.792
SW	**0.047**	**0.964**	**0.128**	**0.048**	**0.957**
UWPropSWA	3.656	0.946	0.126	−4.509	0.283
SW&PropSWA	2.169	0.964	0.138	−4.666	0.394
UWProgWA	1.191	0.959	0.126	−4.592	0.247
SW&ProgSWA	**0.472**	**0.961**	**0.125**	**0.032**	**0.968**

**Note:**

UW, Unweighted; SW, survey-weighted; PropSWA, propensity score weight-adjusted; ProgWA, prognostic score weight-adjusted; AUC, area under curve; CI, confidence interval; bold highlighted are the values for the two best performed models.

## Discussion

Population-based surveys often involve complex sampling schemes to recruit subjects that include unequal probabilities of selected participants in the surveys. Studies often do not incorporate sampling weights in routine data analysis, leading to biased estimates (Bell et al., 2012; Dwivedi, 2022; Khera et al., 2017). In this study, we highlighted different weighting approaches for adjusting confounders and survey weights for prognostic and diagnostic studies with binary and time-to-event outcomes and provided Stata codes for implementation. In our study, uric acid levels were found to be consistently associated with all-cause mortality, and an adjusted estimated threshold of 7 mg/dL may be used to differentiate individuals at high risk and low risk for mortality, particularly in subjects with age >60 years. We found minimal differences in adjusted effect sizes across methods. However, we observed differences between unweighted and survey-weighted analyses for estimating effect sizes and related accuracy measures, particularly with four categories of uric acid concentrations. In addition, our simulation studies identified conditions in which unweighted analyses can produce biased estimates, suggesting that ignoring survey weights in the analysis may lead to biased effect sizes, rAUC, pAUC, and c-statistic. Furthermore, the differences between different weighted analyses, including PropSWA, ProgSWA, and simple adjusted models, indicate that a different approach should be used for covariate adjustment as per the study objective (Dwivedi, 2022) and as recommended in Fig. 2.

Based on NHANES 2005–2018, our study consistently demonstrated uric acid levels as a potential marker for predicting all-cause mortality, particularly among elderly subjects. Similar to our study findings, some studies reported a positive association of uric acid concentrations with all-cause mortality or cardiovascular mortality (Cheong et al., 2017; Fang & Alderman, 2000; Kikuchi et al., 2022; Konta et al., 2020; Kuo et al., 2012). Two studies found a positive association of uric acid levels with all-cause mortality only in men (Niskanen et al., 2004; Zhao et al., 2013), while another study found the same association for elders, only with an overall U-shaped relationship (Tseng et al., 2018). A study showed a positive association of uric acid levels with all-cause mortality in the hemodialysis population when analyzed as a binary variable with a threshold of 8.2 mg/dL and a negative association when the uric acid was used as a continuous exposure (Latif et al., 2011). Another study reported a J-shaped association of uric acid levels with all-cause mortality in hemodialysis patients (Hsu et al., 2004). Our study, including both categorized and continuous forms of uric acid, demonstrated an adverse effect of higher uric acid levels on all-cause mortality in the entire cohort and elderly subjects. The potential mechanism for this association could be its role in causing inflammation, damaging the blood vessel lining, and enhancing the formation of blood clots, leading to a higher risk of heart attack and stroke, and subsequently all-cause mortality. Higher uric acid levels may contribute to increased mortality, possibly due to other comorbid health conditions like hypertension, diabetes, and kidney disease that increase the risk of mortality (Konta et al., 2020).

Although adjusted effect sizes were not quite different between survey-weighted and unweighted analyses, as observed in another study (Lohr & Liu, 1994), the 95% confidence widths of estimated effect sizes were found to be slightly wider for survey-weighted models. Similar to our study, a simulation study concluded that the effect size might be similar between unweighted and weighted analyses, but the 95% confidence width could be quite different between analyses (Oremus et al., 2022). In our study, the confidence widths were quite different in weighted analyses compared to unweighted analyses, depending on the type of analyses, indicating that conclusions can be different between unweighted and weighted analyses (Oremus et al., 2022). In our study, the rAUC and c-statistic were also found to be different across different types of weighted association analyses. Therefore, sampling weight should be included in the analyses to produce accurate estimations and conclusions in prognostic and diagnostic models as recommended (Bollen et al., 2016; Korn & Graubard, 1995). In contrast to association models, the performance of predictive models, as measured by rAUC and c-statistic, was unchanged between weighted and unweighted analyses, indicating that the model summary performance of predictive models does not change in unweighted and weighted analyses. Similarly, a study also noted close estimates of rAUC between unweighted and weighted analyses (Wadekar & Reiter, 2024) and provided scenarios when close estimations of rAUC are expected. Despite identical estimation of rAUC in weighted and unweighted analyses as noted in studies (Wadekar & Reiter, 2024; Yao, Li & Graubard, 2015), it is strongly recommended to use the weighted analyses in studies involving a complex sampling scheme.

Our simulation studies and real data analyses confirmed that there are expected differences in the estimates, particularly with skewed continuous, count, ordinal, or unbalanced exposures. In the presence of skewed and count exposures, the ordinary survey-weighted model produced unbiased estimates of the regression coefficient and AUC. Both unweighted and weighted analyses produced similar and unbiased estimates with a binary exposure in our simulation studies. Similarly, we observed no differences in the estimates between unweighted and weighted analyses, regardless of whether the data were continuous or binary, in the uric acid data analysis. As confirmed in our simulation studies with a count exposure, analysis with four categories of uric acid levels produced substantial differences in unweighted and weighted analyses. Our findings from survey-weighted analyses align with the literature, where the extent of the association between uric acid levels and mortality increases with higher uric acid levels (Hsu et al., 2004). In our real uric acid data analysis, we observed some differences between prognostic and propensity score-weighted analyses. Our simulations identified situations when prognostic and propensity score-weighted analyses produce differences in the estimates. As expected, PropSWA yielded worse performance when there was missing data on covariates but produced relatively superior performance in studies with low-prevalence outcomes. In most scenarios, ordinary survey-weighted or survey-weighted ProgSWA produced similar and better performance compared to other models. Considering the flexibility of the positivity assumption under prognostic score analyses compared to propensity score analysis (Nguyen & Debray, 2019) and its superior performance in most situations, we suggest using survey-weighted ProgSWA for validating findings in observational studies.

PRC analyses reflect changes according to the skewness in datasets, in contrast to AUC analyses, regardless of association or predictive models. Accordingly, we observed notable differences in estimated pAUC for different models in the entire cohort analyses or analysis of older individuals only. Boyd et al. showed that precision and recall are related, and there is a possible region where PRC analysis is unachievable. They suggested potential summary measures of pAUC to account for an unachievable PRC region (Boyd et al., 2012). pAUC estimated *via* PRC analysis is a preferable approach in case of skewed or imbalanced datasets that are commonly observed in medical research, as we also observed in our study. However, the interpretation of pAUC depends on the prevalence or incidence of the outcome according to exposure. In the ROC analysis, the estimated rAUC is compared against 50%, while in the PRC analysis, the estimated pAUC is compared against the estimated prevalence or incidence of the outcome. Since the mortality rate was 7.5% in the entire cohort and 27% among older individuals in our study, the estimated pAUC of 12% or 25% was considered weak, as confirmed with rAUC of 55%. However, the estimated pAUC of 55%with rAUC of 89% indicated good performance of the developed predictive models in our study. Although we used asymptotic normal distribution to estimate the 95% CI of AUC estimates, the binomial distribution may be used to estimate a more appropriate 95% CI of AUC.

The estimated cut points in our study vary between unweighted and covariate-adjusted analyses. Although ***rooted*** and ***roctg*** commands in Stata can be used for obtaining an optimal unadjusted cut point for a continuous marker, these methods do not account for survey weight (Gerke & Zapf, 2022; Reichenheim, 2002). On the other hand, the ***rocreg*** command provides an adjusted cut point of a continuous marker without accounting for survey weight (Pepe, 2003). Our proposed approach of ProgSWA for adjusting covariates and accommodating sampling weights provides a more flexible method for estimating adjusted cut points, particularly in diagnostic studies. However, there are other approaches of ProgSWA (Calkins, 2014) that can be used to estimate the cut points in the survey-weighted adjusted analysis as suggested in our study. Although we need to evaluate the differences in methods incorporated in ***rocreg*** command and our proposed approach of ProgSWA analysis, we strongly recommend using the proposed approach for accommodating survey weight while estimating the adjusted cut point based on simulation and real data analyses.

To the best of our knowledge, our study, for the first time, highlighted possible approaches for accommodating complex survey weights and adjustment of potential confounders in prognostic and diagnostic studies by establishing the association between uric acid levels and all-cause mortality. Our study demonstrated the application of different weighted analyses using the NHANES database, which is commonly used for designing population-based prognostic studies in the US (Ale et al., 2024). Our comprehensive data analysis, including multiple sensitivity analyses such as categorized and continuous forms of uric acid levels, restricted analysis to older subjects (age >60 years), and analysis after removing BMI, considering as a potential mediator, confirmed the robustness of findings and consistencies in estimates across different models and analyses. Through intensive simulation and real data analyses, we provided the preferred method for analyzing complex sampling datasets in the context of different forms of exposure. In addition, our study facilitated the use of statistical codes and step-by-step approaches for incorporating sampling weights through survey-weighted regression, as well as propensity and prognostic score analyses in logistic and Cox regression. Aside from these potential strengths, the study has certain limitations. Although our study reported simulation studies to demonstrate the gain in accommodating survey weights in adjusted analyses, our simulations need to be expanded for different sampling schemes as well as for different methods of propensity score and prognostic score weights. Although we did not observe any mediator in the uric acid data analysis, propensity and prognostic scores should be created with confounders or covariates that do not involve a mediator. In addition, the assumptions related to inverse probability weighting should be tested before finalizing the prognostic or propensity score weights through sensitivity analyses, which involve evaluating changes in effect sizes and balance in the distribution of covariates. The structured algorithms provided in this study can be extended for continuous, multinomial, and ordinal outcomes. In addition, the extension of these methods needs to be established for analyzing longitudinal complex survey datasets. Our study included a single measurement of uric acid that may limit our understanding of its variability and its association with mortality in adults. Despite these limitations, our study shows the possible differences in findings due to ignorance of survey weight and adjustment of covariates while estimating the effect size, model performance measures, and the cut point estimation for continuous exposure. The study also provided evidence that a high level of uric acid was associated with increased mortality and suggested a data-driven adjusted cut point, which may be helpful for researchers in future studies.

## Conclusion

We facilitated a step-by-step illustration of different methods for estimating adjusted effect size and performance measures of diagnostic and prognostic models for a binary and time-to-event outcome after adjusting for covariates with or without accounting for survey weights in Stata. We also reported the application of propensity or prognostic score methods for a continuous or binary exposure in the context of data analysis of complex sampling designs in Stata using real and simulation-based data analyses. The applicability of algorithms was investigated to assess the association between uric acid levels and all-cause mortality in US adults using a population-based NHANES database. Our study findings suggest that ignoring weight for estimating effect size and non-adjusted cut points may produce inaccurate estimates compared to adjusted and weighted analyses. The study also provided evidence that a high level of uric acid was associated with increased mortality, indicating proper screening and management of hyperuricemia, especially in elderly subjects. Based on intensive simulation studies and real data analysis, we strongly recommend incorporating weights while analyzing studies involving complex sampling designs. We also provided preferred methods of incorporating sampling weights while analyzing data from complex sampling designs.

## Supplemental Information

10.7717/peerj.20815/supp-1 Supplemental Information 1 Flowchart for summarizing various primary and sensitivity analyses of uric acid data.

10.7717/peerj.20815/supp-2 Supplemental Information 2 Receiver operating characteristic curve obtained using survey-weighted models for the entire cohort and subpopulation with age>60 years.

10.7717/peerj.20815/supp-3 Supplemental Information 3 Supplemental tables.

10.7717/peerj.20815/supp-4 Supplemental Information 4 Raw data.

10.7717/peerj.20815/supp-5 Supplemental Information 5 Data codebook.

10.7717/peerj.20815/supp-6 Supplemental Information 6 Analytical codes.

## Additional Information and Declarations

### Data Availability

The following information was supplied regarding data availability:

The data and code are available in the Supplemental Files.

The NHANES dataset is available at CDC.gov: https://wwwn.cdc.gov/nchs/nhanes/default.aspx.
