# Supplementary figures and images for: Adjusted effect size, area under the curve, and c-statistic for evaluating the association between uric acid and mortality in US adults using unweighted and survey-weighted regression, propensity, and prognostic score
Source: PeerJ. 2026 Feb 19;14:e20815. doi: 10.7717/peerj.20815 (PMC12925409; doi:10.7717/peerj.20815)

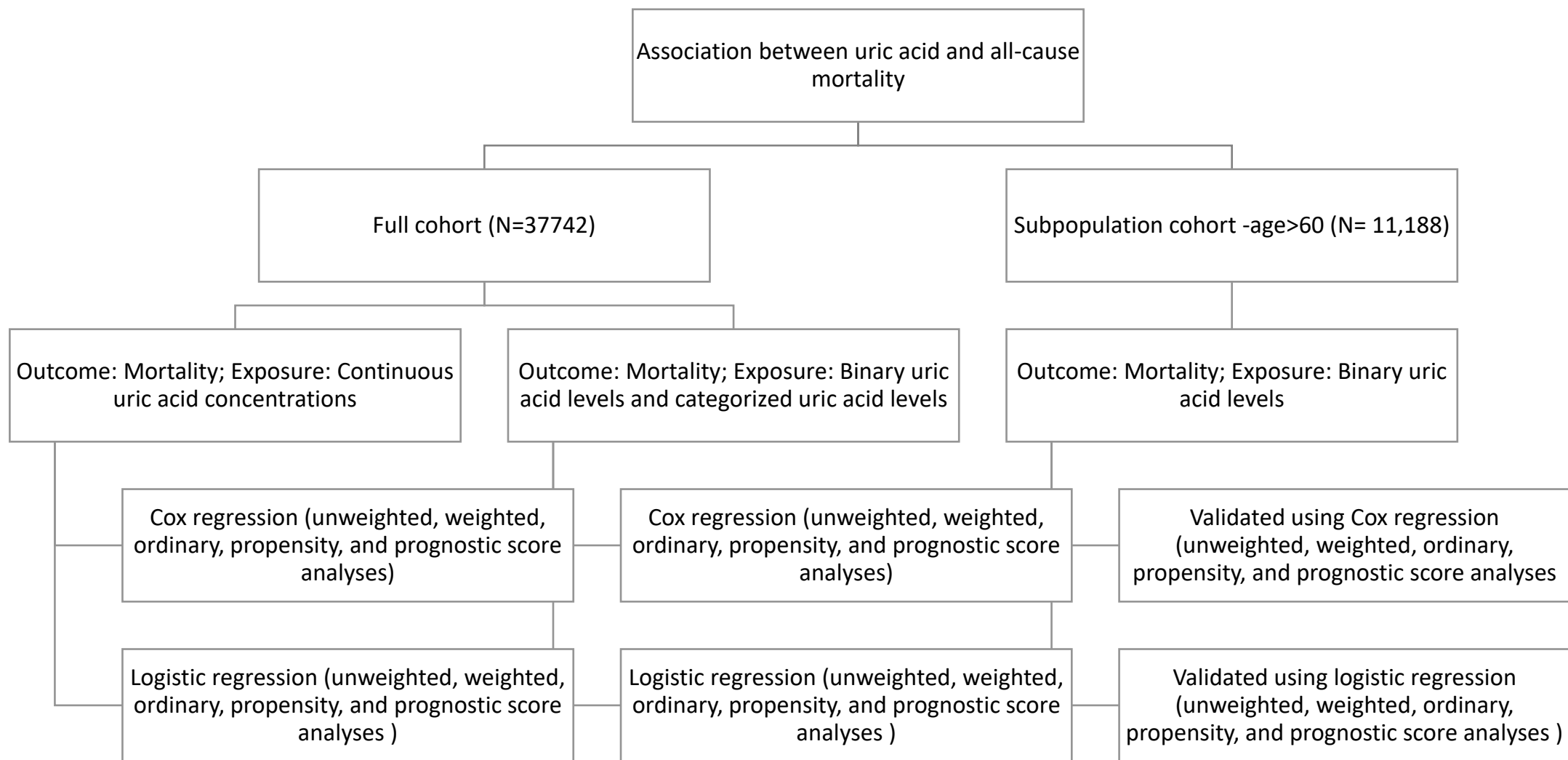

Supplement: Supplemental Information 1 [file peerj-14-20815-s001.pdf]

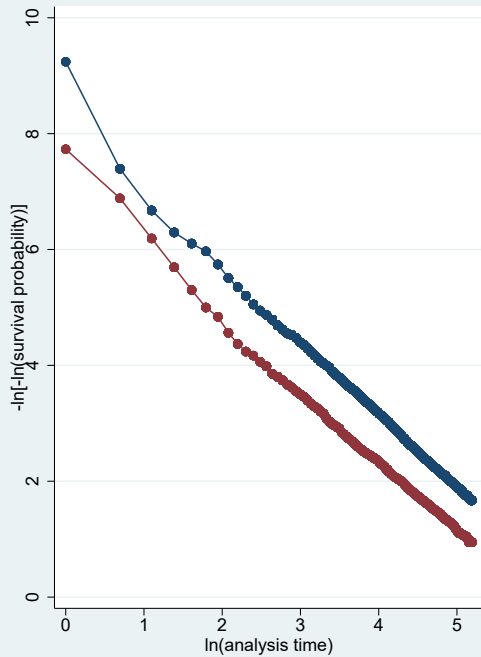

—●— No Hyperuricemia —●— Hyperuricemia

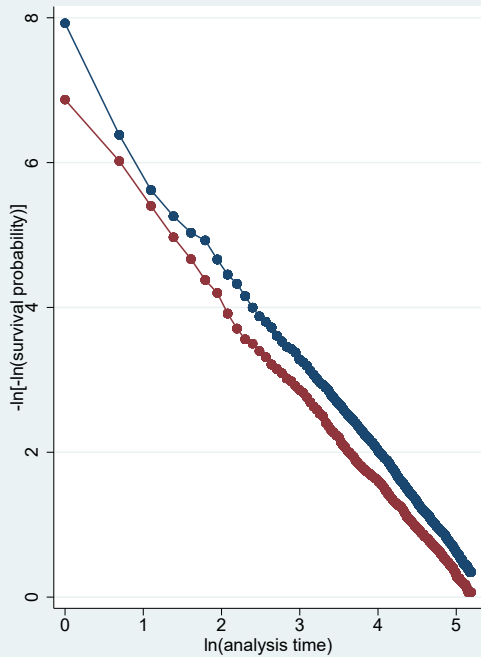

—●— No Hyperuricemia —●— Hyperuricemia

Supplement: Supplemental Information 2 [file peerj-14-20815-s002.pdf]
