# Supplementary material for: Adjusted effect size, area under the curve, and c-statistic for evaluating the association between uric acid and mortality in US adults using unweighted and survey-weighted regression, propensity, and prognostic score
Source: PeerJ. 2026 Feb 19;14:e20815. doi: 10.7717/peerj.20815 (PMC12925409; doi:10.7717/peerj.20815)
Supplement: Supplemental Information 3 [file peerj-14-20815-s003.docx]

**Table S1: Distribution of baseline covariates**

| **Covariates** | **Categories** | **N/mean** | **Percentage/SD** |
| --- | --- | --- | --- |
| Age | Continuous, Mean, Standard Deviation(SD) | 47.9 | 18.7 |
| Income | less than $45000 | 17,039 | 46.6 |
|  | $45000 to $99,999 | 8,279 | 22.7 |
|  | $100,000 or above | 4,751 | 13.0 |
|  | unknown | 7673 | 20.3 |
| Ethnicity | Hispanic | 9834 | 14.4 |
|  | non-Hispanic white | 15703 | 67.1 |
|  | non-Hispanic black | 7966 | 10.9 |
|  | other racial groups | 4239 | 7.7 |
| Sex | male | 18,352 | 48.6 |
|  | female | 19,390 | 51.4 |
| Education | lower than high school | 9,727 | 25.8 |
|  | high school diploma or equivalent | 8,897 | 23.6 |
|  | college or AA degree | 11,008 | 29.2 |
|  | above college qualification | 8,068 | 21.4 |
|  | unknown | 42 | 0.1 |
| Marital Status | never married | 6,797 | 18.0 |
|  | married | 18,497 | 49.0 |
|  | living with a partner | 2,932 | 7.8 |
|  | other categories | 7,929 | 21.0 |
|  | unknown | 1,587 | 4.2 |
| Body Mass Index (kg/m^2^) | <25 | 11,211 | 29.7 |
|  | 25 to 30 | 12,159 | 32.2 |
|  | 30 to 35 | 7,680 | 20.4 |
|  | above 35 | 6,190 | 16.4 |
|  | unknown | 502 | 1.3 |
| Smoking Status | non-smoker | 20,441 | 54.2 |
|  | smoker | 15,989 | 42.4 |
|  | unknown | 1,312 | 3.5 |
| Alcohol Usage | non-alcohol user | 4,991 | 13.2 |
|  | alcohol user | 28,702 | 76.1 |
|  | unknown | 4,049 | 10.7 |
| Physical Activity | no | 11,003 | 29.2 |
|  | yes | 21,763 | 57.7 |
|  | unknown | 4,976 | 13.2 |

**Table S2: Association between uric acid concentrations and all-cause mortality without adjusting for BMI (NHANES 2005-2018)**

|  | **Cox regression** | | **Logistic regression** | | |
| --- | --- | --- | --- | --- | --- |
| **Association models** | **HR [95% CI]** | **p-value** | | **OR [95% CI]** | **p-value** |
| Unweighted & adjusted analysis | 1.08[1.05,1.10] | <0.0001 | | 1.11[1.08,1.14] | <0.0001 |
| Survey-weighted adjusted analysis | 1.08[1.04,1.12] | <0.0001 | | 1.10[1.06,1.14] | <0.0001 |
| Unweighted PropSWA analysis | 1.10[1.05,1.15] | <0.0001 | | 1.11[1.05,1.17] | 0.0004 |
| Survey-weighted & PropSWA analysis | 1.10[1.04,1.15] | 0.0003 | | 1.11[1.05,1.17] | 0.0002 |
| Unweighted ProgSWA | 1.10[1.06,1.13] | <0.0001 | | 1.12[1.08,1.16] | <0.0001 |
| Survey-weighted & ProgSWA analysis | 1.09[1.04,1.13] | 0.0001 | | 1.12[1.06,1.18] | 0.0002 |

Adjusted models included age, gender, income, education, ethnicity, marital status, smoking, alcohol use, and physical activity; gender was not included in creating propensity score weight but was adjusted in regression analysis; age was not included in creating prognostic score weight but was adjusted in regression analysis. PropSWA: Propensity score weight-adjusted; ProgSWA: Prognostic score weight-adjusted; OR: Odds ratio; HR: Hazard ratio; CI: Confidence interval; ^#^ Uric acid was used as continuous exposure in PropSWA analysis; BMI: body mass index

**Table S3: Association between hyperuricemia and all-cause mortality (NHANES 2005-2018)**

|  | **Cox regression** | | **Logistic regression** | |
| --- | --- | --- | --- | --- |
| **Association models** | **HR [95% CI]** | **p-value** | **OR [95% CI]** | **p-value** |
| Unweighted & adjusted analysis | 1.34[1.24,1.44] | <0.0001 | 1.48[1.35,1.63] | <0.0001 |
| Survey-weighted adjusted analysis | 1.35[1.22,1.50] | <0.0001 | 1.45[1.29,1.63] | <0.0001 |
| Unweighted PropSWA analysis | 1.46[1.35,1.59] | <0.0001 | 1.50[1.38,1.64] | <0.0001 |
| Survey-weighted & PropSWA analysis | 1.45[1.30,1.61] | <0.0001 | 1.43[1.28,1.61] | <0.0001 |
| Unweighted ProgSWA analysis | 1.32[1.19,1.47] | <0.0001 | 1.36[1.19,1.55] | <0.0001 |
| Survey-weighted & ProgSWA analysis | 1.28[1.11,1.47] | 0.0011 | 1.31[1.10,1.55] | 0.0023 |

Adjusted models included age, gender, income, education, ethnicity, marital status, body mass index, smoking, alcohol use, and physical activity; gender was not included in creating propensity score weight but was adjusted in regression analysis; age was not included in creating prognostic score weight but was adjusted in regression analysis. PropSWA: Propensity score weight-adjusted; ProgSWA: Prognostic score weight-adjusted; OR: Odds ratio; HR: Hazard ratio; CI: Confidence interval.

**Table S4: Association between uric acid levels (Categorical) and all-cause mortality (NHANES 2005-2018)**

|  | **Cox regression** | | **Logistic regression** | |
| --- | --- | --- | --- | --- |
| **Association models** | **HR [95% CI]** | **p-value** | **OR [95% CI]** | **p-value** |
| Unweighted & adjusted analysis |  |  |  |  |
| 2^nd^ Quartile vs 1^st^ Quartile | 1.08[0.97,1.19] | 0.1718 | 1.14[1.01,1.29] | 0.0367 |
| 3^rd^ Quartile vs 1^st^ Quartile | 1.03[0.92,1.14] | 0.6470 | 1.09[0.97,1.24] | 0.1622 |
| 4^th^ Quartile vs 1^st^ Quartile | 1.39[1.25,1.54] | <0.0001 | 1.58[1.40,1.78] | <0.0001 |
| Survey-weighted adjusted analysis |  |  |  |  |
| 2^nd^ Quartile vs 1^st^ Quartile | 1.15[1.02,1.30] | 0.0237 | 1.21[1.05,1.40] | 0.0112 |
| 3^rd^ Quartile vs 1^st^ Quartile | 1.04[0.92,1.18] | 0.5117 | 1.10[0.95,1.26] | 0.1915 |
| 4^th^ Quartile vs 1^st^ Quartile | 1.42[1.26,1.61] | <0.0001 | 1.55[1.34,1.80] | <0.0001 |
| Unweighted PropSWA analysis |  |  |  |  |
| 2^nd^ Quartile vs 1^st^ Quartile | 1.10[0.98,1.22] | 0.1057 | 1.10[0.97,1.24] | 0.1255 |
| 3^rd^ Quartile vs 1^st^ Quartile | 1.15[1.03,1.28] | 0.0131 | 1.12[0.99,1.27] | 0.0851 |
| 4^th^ Quartile vs 1^st^ Quartile | 1.61[1.45,1.79] | <0.0001 | 1.57[1.37,1.80] | <0.0001 |
| Survey-weighted & PropSWA analysis |  |  |  |  |
| 2^nd^ Quartile vs 1^st^ Quartile | 1.17[1.05,1.30] | 0.0062 | 1.23[1.09,1.39] | 0.0014 |
| 3^rd^ Quartile vs 1^st^ Quartile | 1.15[1.04,1.28] | 0.0095 | 1.27[1.12,1.44] | 0.0003 |
| 4^th^ Quartile vs 1^st^ Quartile | 1.62[1.45,1.82] | <0.0001 | 1.88[1.60,2.20] | <0.0001 |
| Unweighted ProgSWA |  |  |  |  |
| 2^nd^ Quartile vs 1^st^ Quartile | 1.33[1.08,1.64] | <0.0001 | 1.46[1.21,1.76] | <0.0001 |
| 3^rd^ Quartile vs 1^st^ Quartile | 1.55[1.10,2.19] | <0.0001 | 1.72[1.33,2.21] | <0.0001 |
| 4^th^ Quartile vs 1^st^ Quartile | 1.75[1.47,2.10] | <0.0001 | 2.13[1.82,2.49] | <0.0001 |
| Survey-weighted & ProgSWA analysis |  |  |  |  |
| 2^nd^ Quartile vs 1^st^ Quartile | 1.50[1.14,1.99] | 0.0004 | 1.56[1.23,2.00] | 0.0011 |
| 3^rd^ Quartile vs 1^st^ Quartile | 1.54[1.08,2.20] | 0.0011 | 1.62[1.22,2.17] | <0.0001 |
| 4^th^ Quartile vs 1^st^ Quartile | 1.73[1.37,2.19] | <0.0001 | 1.93[1.58,2.36] | <0.0001 |

Adjusted models included age, gender, income, education, ethnicity, marital status, body mass index, smoking, alcohol use, and physical activity; gender was not included in creating propensity score weight but was adjusted in regression analysis; age was not included in creating prognostic score weight but was adjusted in regression analysis. PropSWA: Propensity score weight-adjusted; ProgSWA: Prognostic score weight-adjusted; OR: Odds ratio; HR: Hazard ratio; CI: Confidence interval.

# **Appendix A (Stata codes)**

**cd "**"C:\data_folder**"**

Use the NAHNES 2005-2018 datasets described in the main text of the manuscript.

use "C:\data_folder\mydata.dta", clear

keep sdmvpsu sdmvstra mortstat permth_int mec4yr uric uric_c age_cat2 ridageyr riagendr income_c education2 ethnicity marital bmi_c smoke ever_alc physical_2

save finaldata, replace use finaldata, clear

/*For sugroup Analysis*/

*drop if ridageyr<60

# **/*Logistic regression analysis*/**

## **/*Unweighted and unadjusted analysis (rAUC)*/**

foreach var in phat0 pos nfpos sens spec fpos ppv auc se_auc ll_auc ul_auc youdenid youdenidmax distance mindistance z p_value {

capture drop `var'

}

svyset sdmvpsu [pw=mec4yr], strata(sdmvstra) singleunit(certainty)

**logistic mortstat uric /*unadjusted effect size of uric acid levels*/**

predict phat0

senspec mortstat phat0, sensitivity(sens) specificity(spec) fpos(fpos) ntpos(pos) nfpos(nfpos)

gen ppv=pos/(pos+nfpos)

integ sens fpos, trapezoid

gen auc=r(integral)

return list

tabulate mortstat, matcell(d)

scalar n1= d[2,1]

scalar n2= d[1,1]

scalar q1=auc/(2-auc)

scalar q2=2*auc^2/(1+auc)

scalar auc2=auc^2

scalar se_auc= sqrt( (auc*(1-auc)+ (n1-1)*(q1-auc2)+(n2-1)*(q2-auc2))/(n1*n2))

gen ll_auc= auc-1.96*se_auc

gen ul_auc= auc+1.96*se_auc

gen z=(auc-0.5)/se_auc

gen p_value=2*(1-normal(abs(z)))

tabstat auc z ll_auc ul_auc p_value

**ploting ROC curve

scatter sens fpos, sort(fpos sens) connect(L)

**/*estimation of cut-point for exposure*/**

gen youdenid= sens-(1-spec)

egen youdenidmax= max(youdenid)

gen distance = sqrt((1-sens)^2 + (1-spec)^2)

egen mindistance = min(distance)

/* Identify cut-off point */

list sens spec youdenidmax distance uric if abs(youdenid -youdenidmax)<0.000001

## **/*Unweighted and unadjusted analysis (pAUC)*/**

foreach var in prc se_prc ll_prc ul_prc z p_value {

capture drop `var'

}

prtab mortstat phat0

prcurve mortstat phat0

gen prc=r(AUC)

integ ppv sens, trapezoid /*un-weighted**********/

scalar prc=r(integral) /****return list*****/

return list

tabulate mortstat, matcell(d)

scalar n1= d[2,1]

scalar n2= d[1,1]

scalar q1=prc/(2-prc)

scalar q2=2*prc^2/(1+prc)

scalar prc2=prc^2

gen se_prc= sqrt( (prc*(1-prc)+ (n1-1)*(q1-prc2)+(n2-1)*(q2-prc2))/(n1*n2))

gen ll_prc= prc-1.96*se_prc

gen ul_prc= prc+1.96*se_prc

gen z=(prc-0.5)/se_prc

gen p_value=2*(1-normal(abs(z)))

tabstat prc z ll_prc ul_prc p_value

## **/*Unweighted and adjusted analysis (rAUC)*/**

foreach var in ll_auc ul_auc auc pos nfpos sens spec fpos ppv phat1 youdenid youdenidmax distance mindistance z p_value {

capture drop `var'

}

**logistic mortstat uric ridageyr riagendr i.income_c i.education2 i.ethnicity i.marital i.bmi_c i.smoke ever_alc i.physical_2 /*adjusted effect size of uric acid levels*/**

predict phat1

senspec mortstat phat1, sensitivity(sens) specificity(spec) fpos(fpos) ntpos(pos) nfpos(nfpos)

gen ppv=pos/(pos+nfpos)

integ sens fpos, trapezoid

gen auc=r(integral)

return list

tabulate mortstat, matcell(d)

scalar n1= d[2,1]

scalar n2= d[1,1]

scalar q1=auc/(2-auc)

scalar q2=2*auc^2/(1+auc)

scalar auc2=auc^2

gen se_auc= sqrt( (auc*(1-auc)+ (n1-1)*(q1-auc2)+(n2-1)*(q2-auc2))/(n1*n2))

gen ll_auc= auc-1.96*se_auc

gen ul_auc= auc+1.96*se_auc

gen z=(auc-0.5)/se_auc

gen p_value=2*(1-normal(abs(z)))

tabstat auc z ll_auc ul_auc p_value

## **/*Unweighted and adjusted analysis (pAUC)*/**

foreach var in prc se_prc ll_prc ul_prc z p_value {

capture drop `var'

}

prtab mortstat phat1

prcurve mortstat phat1

gen prc=r(AUC)

integ ppv sens, trapezoid /*un-weighted**********/

scalar prc=r(integral) /****return list*****/

return list

tabulate mortstat, matcell(d)

scalar n1= d[2,1]

scalar n2= d[1,1]

scalar q1=prc/(2-prc)

scalar q2=2*prc^2/(1+prc)

scalar prc2=prc^2

gen se_prc= sqrt( (prc*(1-prc)+ (n1-1)*(q1-prc2)+(n2-1)*(q2-prc2))/(n1*n2))

gen ll_prc= prc-1.96*se_prc

gen ul_prc= prc+1.96*se_prc

gen z=(prc-0.5)/se_prc

gen p_value=2*(1-normal(abs(z)))

tabstat prc z ll_prc ul_prc p_value

## **/*Survey-weighted and unadjusted analysis (rAUC)*/**

foreach var in wauc se_wauc ll_wauc ul_wauc z p_value pos nfpos sens spec fpos ppv phat1 youdenid youdenidmax distance mindistance z_w p_value phat2 ispec {

capture drop `var'

}

**svy:logistic mortstat uric /*survey-weighted unadjusted effect size of uric acid levels*/**

predict phat2

senspec mortstat phat2 [pweight=mec4yr], sensitivity(sens) specificity(spec) fpos(fpos) ntpos(pos) nfpos(nfpos)

gen ppv= pos/(pos+nfpos)

integ sens fpos, trapezoid

gen wauc=r(integral)

return list

tabulate mortstat, matcell(d)

scalar n1= d[2,1]

scalar n2= d[1,1]

scalar q1=wauc/(2-wauc)

scalar q2=2*wauc^2/(1+wauc)

scalar wauc2=wauc^2

gen se_wauc= sqrt( (wauc*(1-wauc)+ (n1-1)*(q1-wauc2)+(n2-1)*(q2-wauc2))/(n1*n2))

gen ll_wauc= wauc-1.96*se_wauc

gen ul_wauc= wauc+1.96*se_wauc

gen z_w=(wauc-0.5)/se_wauc

gen p_value=2*(1-normal(abs(z_w)))

tabstat wauc z_w ll_wauc ul_wauc p_value

scatter sens fpos, sort(fpos sens) connect(L)

** Add zero-zero to graph

tempfile t1

save `t1'

clear

input spec sens

1 0

end

append using `t1'

gen ispec=1-spec

twoway (scatter sens fpos , sort(sens fpos) connect(L) mlab()) (line sens sens)

twoway (scatter sens ispec , sort(sens ispec) connect(L) mlab()) (line sens sens)

/*cut-point estimation*/

gen youdenid= sens-(1-spec)

egen youdenidmax= max(youdenid)

gen distance = sqrt((1-sens)^2 + (1-spec)^2)

egen mindistance = min(distance)

/* Identify cut-off point */

list sens spec youdenidmax distance uric if abs(youdenid -youdenidmax)<0.000001

/*Weighted and unadjusted (pAUC)*/

foreach var in wprc se_wprc ll_wprc ul_wprc wprc z_w p_value {

capture drop `var'

}

integ ppv sens, trapezoid /*weighted**********/

gen wprc=r(integral) /****return list*****/

return list

tabulate mortstat, matcell(d)

scalar n1= d[2,1]

scalar n2= d[1,1]

scalar q1=wprc/(2-wprc)

scalar q2=2*wprc^2/(1+wprc)

scalar wprc2=wprc^2

gen se_wprc=sqrt((wprc*(1-wprc)+(n1-1)*(q1-wprc2)+(n2-1)*(q2-wprc2))/(n1*n2))

gen ll_wprc= wprc-1.96*se_wprc

gen ul_wprc= wprc+1.96*se_wprc

gen z_w=(wprc-0.5)/se_wprc

gen p_value=2*(1-normal(abs(z_w)))

tabstat wprc z_w ll_wprc ul_wprc p_value

scatter ppv sens, sort(fpos sens) connect(L)

twoway (scatter ppv sens , sort(ppv sens) connect(L) mlab())

## **/*Weighted and adjusted analysis (rAUC)*/**

foreach var in wauc se_wauc ll_wauc ul_wauc z p_value pos nfpos sens spec fpos ppv phat1 youdenid youdenidmax distance mindistance z_w p_value phat2 ispec {

capture drop `var'

}

**svy:logistic mortstat uric ridageyr riagendr i.income_c i.education2 i.ethnicity i.marital i.bmi_c i.smoke ever_alc i.physical_2 /*survey-weighted adjusted effect size of uric acid levels*/**

predict phat2

senspec mortstat phat2 [pweight=mec4yr], sensitivity(sens) specificity(spec) fpos(fpos) ntpos(pos) nfpos(nfpos)

gen ppv= pos/(pos+nfpos)

integ sens fpos, trapezoid

gen wauc=r(integral)

return list

tabulate mortstat, matcell(d)

scalar n1= d[2,1]

scalar n2= d[1,1]

scalar q1=wauc/(2-wauc)

scalar q2=2*wauc^2/(1+wauc)

scalar wauc2=wauc^2

gen se_wauc= sqrt( (wauc*(1-wauc)+ (n1-1)*(q1-wauc2)+(n2-1)*(q2-wauc2))/(n1*n2))

gen ll_wauc= wauc-1.96*se_wauc

gen ul_wauc= wauc+1.96*se_wauc

gen z_w=(wauc-0.5)/se_wauc

gen p_value=2*(1-normal(abs(z_w)))

tabstat wauc z_w ll_wauc ul_wauc p_value

** Add zero-zero to graph

tempfile t1

save `t1'

clear

input spec sens

1 0

end

append using `t1'

gen ispec=1-spec

twoway (scatter sens fpos , sort(sens fpos) connect(L) mlab()) (line sens sens)

twoway (scatter sens ispec , sort(sens ispec) connect(L) mlab()) (line sens sens)

**/*cut-point estimation*/**

gen youdenid= sens-(1-spec)

egen youdenidmax= max(youdenid)

gen distance = sqrt((1-sens)^2 + (1-spec)^2)

egen mindistance = min(distance)

**/* Identify cut-off point */**

list sens spec youdenidmax distance uric if abs(youdenid -youdenidmax)<0.000001

## **/*Survey-weighted and adjusted analysis (pAUC)*/**

foreach var in wprc se_wprc ll_wprc ul_wprc wprc z_w p_value {

capture drop `var'

}

integ ppv sens, trapezoid /*weighted**********/

gen wprc=r(integral) /****return list*****/

return list

tabulate mortstat, matcell(d)

scalar n1= d[2,1]

scalar n2= d[1,1]

scalar q1=wprc/(2-wprc)

scalar q2=2*wprc^2/(1+wprc)

scalar wprc2=wprc^2

gen se_wprc=sqrt((wprc*(1-wprc)+(n1-1)*(q1-wprc2)+(n2-1)*(q2-wprc2))/(n1*n2))

gen ll_wprc= wprc-1.96*se_wprc

gen ul_wprc= wprc+1.96*se_wprc

gen z_w=(wprc-0.5)/se_wprc

gen p_value=2*(1-normal(abs(z_w)))

tabstat wprc z_w ll_wprc ul_wprc p_value

scatter ppv sens, sort(fpos sens) connect(L)

twoway (scatter ppv sens , sort(ppv sens) connect(L) mlab())

**/*Unweighted and survey-weighted and PropSWA analyses with a continuous uric acid*/**

foreach var in iptwt newweight sumofweights newweightr norm_weights mypscore wauc se_wauc ll_wauc ul_wauc z p_value pos nfpos sens spec fpos ppv phat1 youdenid youdenidmax distance mindistance z_w p_value phat3 ispec residual0 residual mean0 sd0 wtresi0 wtresi mean sd stgps weightf gps0 gps  muhat0 sdhat0 muhat sdhat {

capture drop `var'

}

xi: reg  uric ridageyr riagendr i.income_c i.education2 i.ethnicity i.marital i.bmi_c i.smoke ever_alc  i.physical_2

predict residual, resid

predict muhat, xb

sum residual

local sdhat= r(sd)

sum uric

gen gps = (1/(`sdhat'*sqrt(2*_pi))) *  exp( -((uric-muhat)^2)/(2*`sdhat'^2) )

svyset sdmvpsu [pw=mec4yr], strata(sdmvstra) singleunit(certainty)

sum uric

local muhat0 =r(mean)

local sdhat0 =r(sd)

gen gps0 = (1/(`sdhat0'*sqrt(2*_pi))) * exp( -((uric - `muhat0')^2)/(2*`sdhat0'^2) )

gen stgps=gps0/gps

gen newweightr=mec4yr* stgps

svyset sdmvpsu [pw=newweightr], strata(sdmvstra) singleunit(certainty)

**svy: logistic mortstat uric /*survey-weighted and propensity score-adjusted effect size of uric acid levels*/**

**logistic mortstat uric [pw= stgps]/*unweighted propensity score-adjusted effect size of uric acid levels*/**

**/*Survey-weighted and PropSWA analysis with a binary uric acid*/**

foreach var in iptwt newweight sumofweights newweightr norm_weights mypscore wauc se_wauc ll_wauc ul_wauc z p_value pos nfpos sens spec fpos ppv phat1 youdenid youdenidmax distance mindistance z_w p_value phat3 ispec {

capture drop `var'

}

xi: psmatch2 uric_c ridageyr i.riagendr i.income_c i.education2 i.ethnicity i.marital i.bmi_c i.smoke i.ever_alc i.physical_2 , outcome(uric_c) logit

rename _pscore mypscore

gen iptwt=(uric_c/mypscore)+((1-uric_c)/(1-mypscore))

egen sumofweights = total(iptwt)

gen norm_weights = iptwt/sumofweights

gen newweight = norm_weights*mec4yr

gen newweightr= round(newweight)

svyset sdmvpsu [pw=newweightr], strata(sdmvstra) singleunit(certainty)

**svy: logistic mortstat uric_c /*survey-weighted and propensity score-adjusted effect size of uric acid levels*/**

**logistic mortstat uric_c [pw= norm_weights]/*unweighted propensity score-adjusted effect size of uric acid levels*/**

## **/*Survey-weighted and PropSWA analysis (rAUC)*/**

svy:logistic mortstat uric

predict phat3

senspec mortstat phat3 [pweight=newweightr], sensitivity(sens) specificity(spec) fpos(fpos) ntpos(pos) nfpos(nfpos)

gen ppv= pos/(pos+nfpos)

integ sens fpos, trapezoid

gen awauc=r(integral)

return list

tabulate mortstat, matcell(d)

scalar n1= d[2,1]

scalar n2= d[1,1]

scalar q1=awauc/(2-awauc)

scalar q2=2*awauc^2/(1+awauc)

scalar awauc2=awauc^2

gen se_awauc= sqrt((awauc*(1-awauc)+ (n1-1)*(q1-awauc2)+(n2-1)*(q2-awauc2))/(n1*n2))

gen ll_awauc= awauc-1.96*se_awauc

gen ul_awauc= awauc+1.96*se_awauc

gen z_w=(awauc-0.5)/se_awauc

gen p_value=2*(1-normal(abs(z_w)))

tabstat awauc z ll_awauc ul_awauc p_value

/*weighted and PropSWA rAUC*/

foreach var in wprc se_wprc ll_wprc ul_wprc wprc z_w p_value {

capture drop `var'

}

integ ppv sens, trapezoid

gen awprc=r(integral)

return list

tabulate mortstat, matcell(d)

scalar n1= d[2,1]

scalar n2= d[1,1]

scalar q1=awprc/(2-awprc)

scalar q2=2*awprc^2/(1+awprc)

scalar awprc2=awprc^2

gen se_awprc= sqrt( (awprc*(1-awprc)+ (n1-1)*(q1-awprc2)+(n2-1)*(q2-awprc2))/(n1*n2))

gen ll_awprc= awprc-1.96*se_awprc

gen ul_awprc= awprc+1.96*se_awprc

cap drop z_w

cap drop p_value

gen z_w=(awprc-0.5)/se_awprc

gen p_value=2*(1-normal(abs(z_w)))

tabstat awprc z_w ll_awprc ul_awprc p_value

**/*Survey-weighted and ProgSWA analysis*/**

foreach var in var iprtwt newweight1 newweightr1 norm_weights mypscore z p_value pos nfpos sens spec fpos ppv phat1 youdenid youdenidmax distance mindistance phat3 ispec awauc se_awauc ll_awauc ul_awauc uricnew lp_pg_sw p {

capture drop `var'

}

svyset sdmvpsu [pw=mec4yr], strata(sdmvstra) singleunit(certainty)

xi:svy :logistic mortstat i.uric ridageyr riagendr i.income_c i.education2 i.ethnicity4 i.marital i.bmi_c i.smoke i.ever_alc  i.physical_2

predict lp_pg_sw_0, xb

gen lp_pg_sw=lp_pg_sw_0-_b[uric]

gen var_pg_sw=exp(lp_pg_sw)/(1+exp(lp_pg_sw))

summarize mortstat

scalar mean_value = r(mean)

gen ipgtwt_sw=(mortstat*mean_value/var_pg_sw)+((1-mortstat)*(1-mean_value)/(1-var_pg_sw))

gen newweightr1=ipgtwt_sw*mec4yr

svyset sdmvpsu [pw=newweightr1], strata(sdmvstra) singleunit(certainty)

**svy: logistic mortstat uric /*survey-weighted and prognostic score-adjusted effect size of uric acid levels*/**

**logistic mortstat uric [pw= ipgtwt_sw]/*unweighted prognostic score-adjusted effect size of uric acid levels*/**

## **/*Survey-weighted and ProgSWA analysis (rAUC)*/**

svy:logistic mortstat uric

predict phat3

senspec mortstat phat3 [pweight=newweightr1], sensitivity(sens) specificity(spec) fpos(fpos) ntpos(pos) nfpos(nfpos)

gen ppv= pos/(pos+nfpos)

integ sens fpos, trapezoid

gen awauc=r(integral)

return list

tabulate mortstat, matcell(d)

scalar n1= d[2,1]

scalar n2= d[1,1]

scalar q1=awauc/(2-awauc)

scalar q2=2*awauc^2/(1+awauc)

scalar awauc2=awauc^2

gen se_awauc= sqrt((awauc*(1-awauc)+ (n1-1)*(q1-awauc2)+(n2-1)*(q2-awauc2))/(n1*n2))

gen ll_awauc= awauc-1.96*se_awauc

gen ul_awauc= awauc+1.96*se_awauc

gen z_w=(awauc-0.5)/se_awauc

gen p_value=2*(1-normal(abs(z_w)))

tabstat awauc z ll_awauc ul_awauc p_value

**/*Estimation of cut-point*/**

gen youdenid= sens-(1-spec)

egen youdenidmax= max(youdenid)

gen distance = sqrt((1-sens)^2 + (1-spec)^2)

egen mindistance = min(distance)

**/* Identify cut-off point */**

list sens spec youdenidmax distance uric if abs(youdenid -youdenidmax)<0.000001

/*weighted and ProgSWA(or MPropSWA) pAUC*/

foreach var in awprc se_awprc ll_awprc ul_awprc z_w p_value {

capture drop `var'

}

integ ppv sens, trapezoid

gen awprc=r(integral)

return list

tabulate mortstat, matcell(d)

scalar n1= d[2,1]

scalar n2= d[1,1]

scalar q1=awprc/(2-awprc)

scalar q2=2*awprc^2/(1+awprc)

scalar awprc2=awprc^2

gen se_awprc= sqrt( (awprc*(1-awprc)+ (n1-1)*(q1-awprc2)+(n2-1)*(q2-awprc2))/(n1*n2))

gen ll_awprc= awprc-1.96*se_awprc

gen ul_awprc= awprc+1.96*se_awprc

gen z_w=(awprc-0.5)/se_awprc

gen p_value=2*(1-normal(abs(z_w)))

tabstat awprc z_w ll_awprc ul_awprc p_value

# **/*Cox regression analysis*/**

stset permth_int, failure (mortstat=1)

cap drop censind

generate censind=1-_d if _st==1

## **/*Unweighted and unadjusted analysis (c-statistic)*/**

**stcox uric /*unweighted unadjusted effect size of uric acid levels*/**

cap drop hr

predict hr

cap drop invhr

gen invhr=1/hr

somersd _t invhr if _st==1, cenind(censind) tdist transf(c)

## **/*Unweighted and adjusted analysis (c-statistic)*/**

**stcox uric ridageyr riagendr i.income_c i.education2 i.ethnicity i.marital i.bmi_c i.smoke ever_alc i.physical_2** **/*unweighted adjusted effect size of uric acid levels*/**

cap drop hr

predict hr

cap drop invhr

gen invhr=1/hr

somersd _t invhr if _st==1, cenind(censind) tdist transf(c)

## **/*Survey-weighted and unadjusted analysis (c-statistic)*/**

svyset sdmvpsu [pw=mec4yr], strata(sdmvstra) singleunit(certainty)

**svy: stcox uric /*survey-weighted unadjusted effect size of uric acid levels*/**

cap drop hr

predict hr

cap drop invhr

gen invhr=1/hr

somersd _t invhr [pw=mec4yr] if _st==1, cenind(censind) tdist transf(c)

## **/*Survey-weighted and adjusted analysis (c-statistic)*/**

svyset sdmvpsu [pw=mec4yr], strata(sdmvstra) singleunit(certainty)

**svy: stcox uric ridageyr riagendr i.income_c i.education2 i.ethnicity i.marital i.bmi_c i.smoke ever_alc i.physical_2 /*survey-weighted adjusted effect size of uric acid levels*/**

cap drop hr

predict hr

cap drop invhr

gen invhr=1/hr

somersd _t invhr [pw=mec4yr] if _st==1, cenind(censind) tdist transf(c)

## **/*Unweighted and survey-weighted and PropSWA analysis (c-statistic)*/**

svyset sdmvpsu [pw=stgps], strata(sdmvstra) singleunit(certainty)

stset permth_int, failure (mortstat=1)

**svy: stcox uric /*unweighted and propensity score-adjusted effect size of continuous uric acid levels*/**

svyset sdmvpsu [pw=norm_weights], strata(sdmvstra) singleunit(certainty)

stset permth_int, failure (mortstat=1)

**svy: stcox uric_c /*unweighted and propensity score-adjusted effect size of binary uric acid levels*/**

svyset sdmvpsu [pw=newweightr], strata(sdmvstra) singleunit(certainty)

stset permth_int, failure (mortstat=1)

*****************************************************

cap drop censind

generate censind=1-_d if _st==1

**svy: stcox uric /*survey-weighted and propensity score-adjusted effect size of continuous uric acid levels*/**

cap drop hr

predict hr

cap drop invhr

gen invhr=1/hr

somersd _t invhr [pw=newweightr] if _st==1, cenind(censind) tdist transf(c)

## **/*Unweighted and survey-weighted and ProgSWA analysis (c-statistic)*/**

svyset sdmvpsu [pw= ipgtwt_sw], strata(sdmvstra) singleunit(certainty)

stset permth_int, failure (mortstat=1)

**svy: stcox uric_c /*unweighted and propensity score-adjusted effect size of binary uric acid levels*/**

**svy: stcox uric /*unweighted and propensity score-adjusted effect size of continuous uric acid levels*/**

svyset sdmvpsu [pw=newweightr1], strata(sdmvstra) singleunit(certainty)

stset permth_int, failure (mortstat=1)

*******************************************

**svy: stcox uric /*survey-weighted and prognostic score-adjusted effect size of uric acid levels*/**

cap drop hr

predict hr

cap drop invhr

gen invhr=1/hr

somersd _t invhr [pw=newweightr1] if _st==1, cenind(censind) tdist transf(c)
