# Supplementary material for: Adjusted effect size, area under the curve, and c-statistic for evaluating the association between uric acid and mortality in US adults using unweighted and survey-weighted regression, propensity, and prognostic score
Source: PeerJ. 2026 Feb 19;14:e20815. doi: 10.7717/peerj.20815 (PMC12925409; doi:10.7717/peerj.20815)
Supplement: Supplemental Information 5 [file peerj-14-20815-s005.docx]

| **Variables** | **Labels** | **Code** |
| --- | --- | --- |
| sdmvpsu | primary sampling unit |  |
| mec4yr | survey weight |  |
| sdmvstra | strata |  |
| mortstat | Mortality | 1= death and 0 alive |
| permth_int | time to mortality |  |
| uric | uric acid levels |  |
| uric_c | binary uric acid | 1; high and 0 low |
| uric_cat1 | four categories of uric acid | 4 quartiles |
| ridageyr | age |  |
| age3 | age | 3 tertiles |
| income_c | less than $45000 | 1 |
|  | $45000 to $99,999 | 2 |
|  | $100,000 or above | 3 |
|  | unknown | 99 |
| ethnicity4 | Hispanic | 1 |
|  | non-Hispanic white | 2 |
|  | non-Hispanic black | 3 |
|  | other racial groups | 4 |
| riagendr | male | 1 |
|  | female | 2 |
| education2 | lower than high school | 1 |
|  | high school diploma or equivalent | 2 |
|  | college or AA degree | 3 |
|  | above college qualification | 4 |
|  | unknown | 99 |
| marital | never married | 0 |
|  | married | 1 |
|  | living with a partner | 2 |
|  | other categories | 3 |
|  | unknown | 99 |
| bmi_c | <25 | 1 |
|  | 25 to 30 | 2 |
|  | 30 to 35 | 3 |
|  | above 35 | 4 |
|  | unknown | 99 |
| smoke | non-smoker | 0 |
|  | smoker | 1 |
|  | unknown | 99 |
| ever_alc | non-alcohol user | 0 |
|  | alcohol user | 1 |
|  | unknown | 99 |
| physical_2 | no | 0 |
|  | yes | 1 |
|  | unknown | 99 |
